# The Effects of Separate and Combined Treatment of Male Rats with Type 2 Diabetes with Metformin and Orthosteric and Allosteric Agonists of Luteinizing Hormone Receptor on Steroidogenesis and Spermatogenesis

**DOI:** 10.3390/ijms23010198

**Published:** 2021-12-24

**Authors:** Andrey A. Bakhtyukov, Kira V. Derkach, Viktor N. Sorokoumov, Anna M. Stepochkina, Irina V. Romanova, Irina Yu. Morina, Irina O. Zakharova, Liubov V. Bayunova, Alexander O. Shpakov

**Affiliations:** 1 Sechenov Institute of Evolutionary Physiology and Biochemistry of Russian Academy of Sciences, 194223 St. Petersburg, Russia; bahtyukov@gmail.com (A.A.B.); derkatch_k@list.ru (K.V.D.); sorokoumov@gmail.com (V.N.S.); annastepochkina23.11@mail.ru (A.M.S.); irinaromanova@mail.ru (I.V.R.); irinamorina@mail.ru (I.Y.M.); irinaz6969@mail.ru (I.O.Z.); bayunoval@mail.ru (L.V.B.); 2 Institute of Chemistry, Saint Petersburg State University, 198504 St. Petersburg, Russia

**Keywords:** metformin, male reproduction, type 2 diabetes mellitus, allosteric agonist, luteinizing hormone receptor, chorionic gonadotropin, testicular steroidogenesis, spermatogenesis

## Abstract

In men with type 2 diabetes mellitus (T2DM), steroidogenesis and spermatogenesis are impaired. Metformin and the agonists of luteinizing hormone/human chorionic gonadotropin(hCG)-receptor (LH/hCG-R) (hCG, low-molecular-weight allosteric LH/hCG-R-agonists) can be used to restore them. The aim was to study effectiveness of separate and combined administration of metformin, hCG and 5-amino-*N-tert*-butyl-2-(methylsulfanyl)-4-(3-(nicotinamido)phenyl)thieno[2,3-*d*]pyrimidine-6-carboxamide (TP3) on steroidogenesis and spermatogenesis in male rats with T2DM. hCG (15 IU/rat/day) and TP3 (15 mg/kg/day) were injected in the last five days of five-week metformin treatment (120 mg/kg/day). Metformin improved testicular steroidogenesis and spermatogenesis and restored LH/hCG-R-expression. Compared to control, in T2DM, hCG stimulated steroidogenesis and StAR-gene expression less effectively and, after five-day administration, reduced LH/hCG-R-expression, while TP3 effects changed weaker. In co-administration of metformin and LH/hCG-R-agonists, on the first day, stimulating effects of LH/hCG-R-agonists on testosterone levels and hCG-stimulated expression of StAR- and CYP17A1-genes were increased, but on the 3–5th day, they disappeared. This was due to reduced LH/hCG-R-gene expression and increased aromatase-catalyzed estradiol production. With co-administration, LH/hCG-R-agonists did not contribute to improving spermatogenesis, induced by metformin. Thus, in T2DM, metformin and LH/hCG-R-agonists restore steroidogenesis and spermatogenesis, with metformin being more effective in restoring spermatogenesis, and their co-administration improves LH/hCG-R-agonist-stimulating testicular steroidogenesis in acute but not chronic administration.

## 1. Introduction

Biguanide metformin (MF), a first-line drug for the treatment of type 2 diabetes mellitus (T2DM), is widely used to normalize insulin sensitivity and improve glucose and lipid metabolism in patients with T2DM and metabolic syndrome [1,2,3,4]. In diabetic pathology, there is numerous evidence that MF also improves the functions of the impaired endocrine system, including the restoration of hypothalamic-pituitary-gonadal (HPG) axis, both in men and women [5,6,7,8,9]. This is due to both the normalization of hypothalamic regulation of the endocrine system, since the hypothalamus is one of the targets of MF, which easily penetrates the blood-brain barrier [10,11], and the beneficial effect of MF therapy on metabolism and hormonal regulation at the periphery, including the testes and ovaries [12,13,14,15,16,17]. In male diabetic patients and in animals with experimental models of diabetes, long-term MF therapy improves the spermatogenesis, increasing the fertilizing ability of sperm [7,8,9,18,19].

It is well known that luteinizing hormone (LH) and human chorionic gonadotropin (hCG), its structural and functional homologue, are the main regulators of steroidogenesis in testicular Leydig cells [20,21,22]. LH and hCG bind with a high affinity to the orthosteric site located in the ectodomain of G protein-coupled LH/hCG receptor (LH/hCG-R). This binding leads to conformational rearrangements in the extracellular loops and the transmembrane domain of LH/hCG-R and induces the activation of heterotrimeric G proteins (G_s_, G_q/11_) and β-arrestins. As a result, the intracellular signaling cascades are activated, which are responsible for the regulation of testosterone synthesis, as well as for the maturation and survival of testicular cells [20,21,22,23]. Testosterone secretion by Leydig cells makes a major contribution to the control of sperm maturation and male fertility [24,25,26]. However, the use of gonadotropins in the clinic for the treatment of hypogonadotropic hypogonadism, delayed puberty in boys and age-related androgen deficiency often leads to side effects [27,28,29]. This may be due to structural differences between the gonadotropin preparations (recombinant LH, recombinant and urinary hCG), which changes the effectiveness of their stimulating effect on various intracellular cascades and effector proteins [28]. There is evidence that pharmacological preparations of gonadotropins are characterized by immunogenicity, and their long-term use can lead to resistance of the testes to endogenous gonadotropins, due to the triggering of the mechanisms of LH/hCG-R desensitization [21,22,30,31,32,33].

These limitations require the development of new regulators of LH/hCG-R activity. In this regard, low-molecular-weight allosteric LH/hCG-R-agonists, which interact with an allosteric site located in the transmembrane domain of LH/hCG-R, are of considerable interest [34]. Among allosteric agonists, thieno[2,3-*d*]-pyrimidine derivatives, developed in 2002 by Dutch scientists [35], demonstrated a high efficiency [36,37,38]. We further developed thieno[2,3-*d*]-pyrimidine derivatives 5-amino-*N-tert*-butyl-2-(methylsulfanyl)-4-(3-(nicotinamido)phenyl)thieno[2,3-*d*] pyrimidine-6-carboxamide (TP3) (Figure S1) and its analogues TP1 and TP4/2, which showed significant steroidogenic activity in the in vitro and in vivo conditions [38,39,40]. They regulated the testosterone production in aging rats and animals with streptozotocin (STZ) diabetes [38,41,42,43]. Thieno[2,3-*d*]-pyrimidine derivatives selectively stimulated the enzyme adenylyl cyclase, increasing the level of intracellular cAMP and activating protein kinase A [38,40,44]. This resulted in the activation of steroidogenic acute regulatory protein (StAR), a mitochondrial cholesterol-transporting protein, which catalyzes the first, rate-limiting stage of steroidogenesis [45]. The consequence of StAR activation is the triggering steroidogenic reactions leading to the synthesis of pregnenolone (cytochrome P450_scc_, CYP11A1), progesterone (3β-hydroxysteroid dehydrogenase/Δ5-4 isomerase), 17-hydroxyprogesterone, androstenedione (cytochrome P450 17A1/steroid 17α-monooxygenase, CYP17A1) and testosterone (17β-hydroxysteroid dehydrogenase) [46,47,48,49].

Since MF improves metabolic and hormonal indices and testicular morphology in T2DM, there is reason to believe that the testicular response to LH/hCG-R-agonists, reduced in diabetic pathology, can increase with MF treatment. This is also supported by our data that MF improves the stimulation of testosterone production caused by a single injection of hCG [50]. However, the effect of MF therapy on the steroidogenic and spermatogenic effects of hCG, which is injected into diabetic animals for several days, has not yet been studied. Moreover, there are no data on the MF influence on the steroidogenic and spermatogenic effects of low-molecular-weight LH/hCG-R-agonists. A comparative study of the improving effect of MF therapy, orthosteric and allosteric LH/hCG-R-agonists and their combinations on steroidogenesis and spermatogenesis is very important for developing an optimal strategy for normalizing reproductive functions in T2DM, as well as for increasing the effectiveness of assisted reproductive technology in diabetic pathology.

Thus, the aim of this work was to study the effects of five-week MF therapy and five-day treatment with allosteric (TP3) or orthosteric (hCG) LH/hCG-R-agonists, as well as their combination (in the fifth week of MF treatment) on the testicular steroidogenesis and spermatogenesis in rats with a high-fat diet (HFD)/STZ-induced T2DM. As a result, we have shown for the first time that in T2DM rats treated with MF, the steroidogenic effects of single-dose orthosteric and allosteric LH/hCG-R-agonists, which differ in their mechanisms of receptor activation, are enhanced. This is illustrated by an increase in testosterone production, and in the case of hCG, an increase in the testicular expression of steroidogenic genes (transport protein StAR, cytochrome CYP17A1). These effects are accompanied by the cancellation of LH/hCG-R-agonist-induced stimulating effect on testicular expression of the aromatase gene, which was demonstrated in control and diabetic rats, as well as the maintenance of normal expression of the gene encoding LH/hCG-R. In turn, after a five-day administration of LH/hCG-R-agonists, their testosterone-stimulating effect is suppressed, which is associated with a significant decrease in LH/hCG-R expression in the testes, as well as an increase in estradiol production in comparison with a one-day LH/hCG-R-agonists administration. The data obtained significantly change the strategy for the possible use of complex therapy with MF (and, possibly, other antidiabetic drugs) and LH/hCG-R-agonists (and, possibly, other activators of steroidogenesis) to compensate for impaired steroidogenesis and spermatogenesis in T2DM and indicate the need for further research in this areas, including clinical. We have shown for the first time that in T2DM, the steroidogenic effects of the allosteric LH/hCG-R-agonist TP3 are more stable as compared to hCG, including with prolonged administration, and this makes the use of allosteric LH/hCG-R-agonists very promising for mild, long-term correction of androgen deficiency in T2DM. This is consistent with our early data on the maintenance of the steroidogenic effect of another LH/hCG-R-agonist, TP4/2, when it is used to restore androgen deficiency in T1DM [38].

## 2. Results

### 2.1. Characterization of T2DM Model in Male Rats, and the Effect of Treatment with Metformin and LH/hCG-R-Agonists

Male rats with T2DM had an increase in the body weight, the increased levels of glucose, glycated hemoglobin (HbA1c), insulin, leptin, triglycerides and total cholesterol, and an increase in the insulin resistance index (Table 1). Four-week treatment of diabetic animals with MF (120 mg/kg/day) led to a decrease in the body weight, normalization of the plasma levels of glucose, HbA1c, insulin, leptin and lipids and an improvement of insulin sensitivity (Table 1). Diabetic rats had a lower baseline testosterone level, and it was partially restored with MF treatment (Table 1).

To study the effect of LH/hCG-R-agonists on the control, diabetic and MF-treated diabetic rats, 18 experimental groups of animals were formed (nine groups for studying a single administration of LH/hCG-R-agonists, and nine groups for studying a five-day administration of LH/hCG-R-agonists) (for a detailed description of the groups and the design of experiments, see Section 4.3).

In both control and diabetic animals, five-day treatment with TP3 and hCG did not significantly affect the body and fat weights and the blood levels of glucose, HbA1c, insulin, leptin and lipids. In MF-treated diabetic rats, LH/hCG-R-agonists also did not contribute to improving effects of MF on the body and fat weights and the blood levels of glucose, HbA1c, insulin and leptin (Tables S1 and S2). In hCG-treated groups, there was a tendency for an increase in the testes weight, but the difference with the untreated groups was not significant (Table S1). In the CG5 group, the gonadosomatic index (GSI), which was measured as (testicular weight/total body weight) × 100, was significantly increased as compared to control animals (Table S1). Meanwhile, in the DM5 group, the GSI was higher than in the untreated diabetic rats, which is due to a MF-induced decrease in the body weight, and as a consequence, an increase in the testes/body weight ratio (Table S1).

It should be noted that the compound TP3, when administered to male rats (15 mg/kg, i.p.), was stable in the blood (half-life was at least 6 h), as indicated by our studies on the dynamics of the concentration of this compound in the blood (Table S3). In addition, according to our data on the distribution of TP3 in the rat tissues 6 h after administration, this compound accumulated in comparable amounts in the testes, thyroid gland and liver (Table S4). In smaller amounts, the TP3 was found in the brain (Table S4). These results indicate the stability and bioavailability of TP3 for Leydig cells, its main target, when this drug administered intraperitoneally.

### 2.2. Effects of Metformin and LH/hCG-R-Agonists and Their Combination on the Blood Testosterone Levels in Control and Diabetic Rats

**Figure 1:**
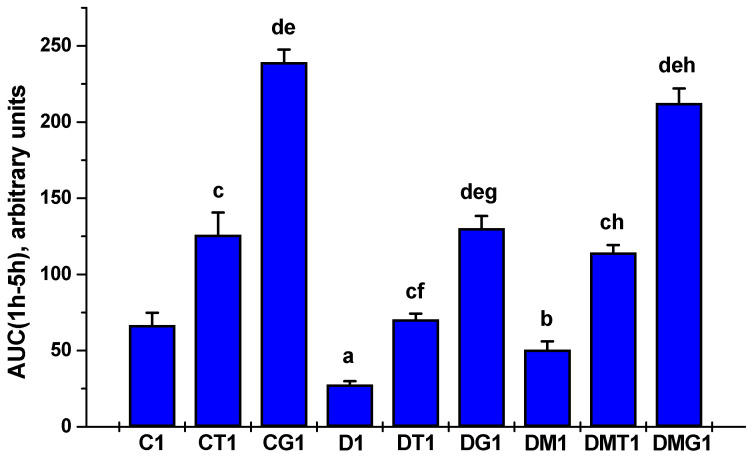
The AUC_1h-5h_ values for the curves “testosterone concentration (nM) vs. time (hours)” in Table 3. and hCG. The AUC_1h-5h_ values are given for the curves “testosterone concentration (nM) vs. time (hours)”, where 1h–5h represent the intervals from the first to fifth hours of treatment. The duration of MF treatment (120 mg/kg/day) was five weeks, and the single doses of TP3 and hCG were 15 mg/kg and 15 IU/rat, respectively. A single injection of LH/hCG-R-agonists was carried out on the 35th day of MF treatment (the final day of the experiment). ^a^—the difference between the C1 vs. D1; ^b^—the difference between the D1 vs. DM1; ^c^—the difference between the C1 vs. CT1, D1 vs. DT1 and DM1 vs. DMT1; ^d^—the difference between the C1 vs. CG1, D1 vs. DG1 and DM1 vs. DMG1; ^e^—the difference between the CT1 vs. CG1, DT1 vs. DG1 and DMT1 vs. DMG1; ^f^—the difference between the CT1 vs. DT1; ^g^—the difference between the CG1 vs. DG1 or DMG1; and ^h^—the difference between the DT1 vs. DMT1 and DG1 vs. DMG1 are significant at *p* < 0.05. The data are presented as the M ± SEM, *n* = 5.

**Figure 2:**
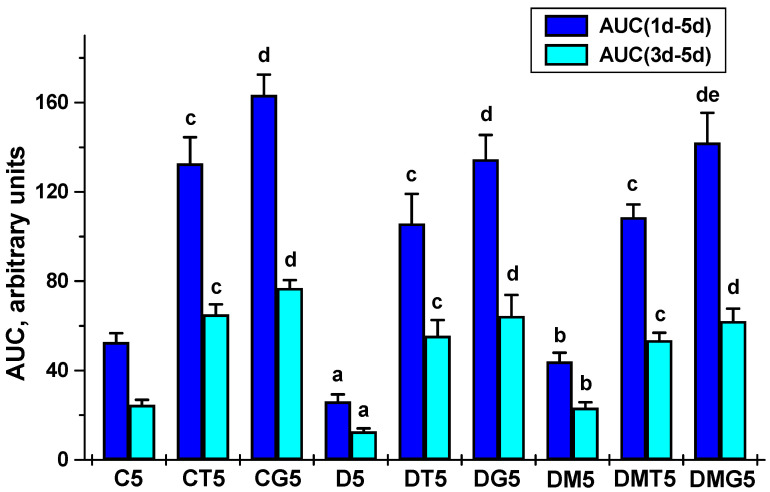
The AUC_1d-5d_ and AUC_3d-5d_ values for the curves “testosterone concentration (nM) vs. time (days)” in the control and diabetic animals, and the effects of five-week metformin treatment and five-day administration of TP3 and hCG. The AUC_1d-5d_ and AUC_3d-5d_ values are given for the curves “testosterone concentration (nM) vs. time (days)”, where 1–5 and 3–5 represent the intervals from the first to fifth days and from the third to fifth days of LH/hCG-R-agonists treatment, respectively. The duration of MF treatment (120 mg/kg/day) was five weeks, and the daily doses of TP3 and hCG were 15 mg/kg and 15 IU/rat, respectively. Five-day treatment with TP3 and hCG was carried out from the 31st to the 35th days of MF treatment. ^a^—the difference between the C5 vs. D5; ^b^—the difference between the D5 vs. DM5; ^c^—the difference between the C5 vs. CT5, D5 vs. DT5 and DM5 vs. DMT5; ^d^—the difference between the C5 vs. CG5, D5 vs. DG5 and DM5 vs. DMG5; and ^e^—the difference between the DMT5 vs. DMG5 are significant at *p* < 0.05. The data are presented as the M ± SEM, *n* = 5.

Testosterone production in T2DM rats was reduced in comparison with the control animals, which is demonstrated by the daily and five-day dynamics of testosterone concentration and the AUC_1h-5h_ and AUC_1d-5d_ values for the “testosterone concentration vs. time (hours or days)” curves (Table 2 and Table 3, Figure 1 and Figure 2). Five-week MF treatment partially restored testosterone production in diabetic rats. This was illustrated by the higher AUC_1h-5h_ and AUC_1d-5d_ in the MF-treated rats as compared to untreated animals (Figure 1 and Figure 2).

In control rats, with a single administration of LH/hCG-R-agonists, the stimulating effect of hCG on testosterone levels significantly exceeded the corresponding effect of TP3 (Table 2). In T2DM, the testosterone-stimulating effects of both LH/hCG-R-agonists were reduced, and in MF-treated diabetic rats, they became more pronounced in comparison with diabetic rats receiving only LH/hCG-R-agonists or only MF (Table 2). This was demonstrated by the AUC_1h-5h_ for TP3 and hCG effects, which in the DMT1 and DMG1 groups were 62% and 63% higher than in the DT1 and DG1 groups (Figure 1). These data indicate that MF treatment resulted in a significant enhancement of the steroidogenic effects of both LH/hCG-R-agonists upon single administration.

In both control and diabetic rats, on the second to fifth days of five-day treatment with LH/hCG-R-agonists, the differences between the testosterone-stimulating effects of hCG and TP3 were not significant, excluding the fourth day in the control groups (Table 3), and the AUC_1d-5d_ and AUC_3d-5d_ for these effects did not differ significantly (Figure 2). In the diabetic groups on the first day of treatment, these effects were weakened as compared to control, but then the differences between the testosterone-stimulating effects of TP3 and hCG in control and diabetic rats were disappeared (Table 3). In the DMT5 and DMG5 groups, despite the improvement in the steroidogenic effects of TP3 and hCG on the first day, on the second to fifth days, the testosterone-stimulating effects of these drugs did not differ from those in the DT5 and DG5 groups, which was illustrated by the AUC_1d-5d_ and AUC_3d-5d_ values (Table 3, Figure 2).

### 2.3. Effects of Metformin and LH/hCG-R-Agonists and Their Combination on the Content of Testosterone, Estradiol and Their Precursors in the Testes of Control and Diabetic Rats

In the testes of control and diabetic rats, the content of progesterone, 17-hydroxyprogesterone and androstenedione, the precursors of testosterone, did not differ significantly, and the MF treatment did not affect them (Table 4 and Table 5). At the same time, the testosterone content in diabetic rats was reduced in comparison with control animals, and MF treatment restored it (Table 4 and Table 5). In the D1 group, the progesterone/testosterone, 17-OH-progesterone/testosterone, and androstenedione/testosterone ratios in the testes were 91, 79 and 122% higher than in control animals, and MF treatment reduced them to control levels (Table S5). Similar results were obtained for these ratios in the groups C5, D5 and DM5 (Table S6).

With a single injection, TP3 had a little effect on the levels of the testosterone and its precursors in control and diabetic rats. Only in the DT1 group, TP3 increased the testosterone content in comparison with the D1 group (Table 4). In the CT1 and DMT1 groups, there was a trend towards an increase in testosterone levels, but the differences with the C1 and DM1 groups were not significant (Table 4). With a single administration to control rats, hCG increased the levels of progesterone and 17-OH-progesterone in comparison with the C1 and CT1 groups, and in the case of testosterone only in relation to the C1 group (Table 4). In the DG1 group, hCG stimulating effects on progesterone and testosterone content were maintained (Table 4). In MF-treated rats, hCG increased the content of testosterone and its precursors. The content of progesterone and 17-OH-progesterone in the DMG1 group was significantly higher than in the DMT1 group, while the content of 17-OH-progesterone and testosterone was higher than in the DG1 group, and in the case of 17-OH-progesterone and androstenedione the difference was significant (Table 4). This indicates an enhancement of the stimulating effect of a single dose of hCG on the production of testosterone and its precursors in the testes of MF-treated rats and is in a good agreement with the data on blood testosterone levels 5 h after a single injection of hCG (Table 2, Figure 1). In addition, a single administration of TP3 and hCG led to a decrease in the progesterone/testosterone, 17-OH-progesterone/testosterone, and androstenedione/testosterone ratios in the control and diabetic rats, including those treated with MF, which indicates an LH/hCG-R-agonist-induced increase in the rate of testicular steroidogenesis reactions (Table S5).

In the testes of diabetic rats, the intratesticular level of estradiol did not differ from that in the control, but it was reduced by MF treatment (Table 4). The testosterone/estradiol ratio was significantly decreased in T2DM and was restored in MF-treated rats (Table S5). In the CG1 and DG1 groups with a single gonadotropin treatment, the estradiol level was decreased significantly, while TP3 considerably reduced the estradiol level only in the DT1 group (Table 4). Both LH/hCG-R-agonists increased the testosterone/estradiol ratio, which was less pronounced in T2DM and most pronounced in the MF-treated groups (Table S5).

After five days of administration, TP3 and hCG increased the intratesticular levels of testosterone and its precursors in both the control and diabetic groups. In the case of androstenedione and testosterone, the differences with the C5 and D5 groups were significant for both LH/hCG-R-agonists, excluding the group CT5 (*p* = 0.052 as compared to control) (Table 5). Meanwhile, in the testes of MF-treated rats, hCG increased the content of all testosterone precursors, but did not affect the testosterone content, while TP3 increased only the androstenedione content. In the MF-treated groups, hCG increased intratesticular progesterone and 17-OH-progesterone levels to a greater extent as compared to TP3, while no differences were found in hCG- and TP3-induced testosterone levels (Table 5). This correlates with blood testosterone levels in rats with five-day LH/hCG-R-agonist treatment (Table 3, Figure 2). In contrast to the one-day administration, in the case of the five-day hCG treatment, the progesterone/testosterone, 17-OH-progesterone/testosterone, and androstenedione/testosterone ratios were changed to a significantly lesser extent, and in the DMG5 group, a significant increase in the ratio of 17-OH-progesterone/testosterone, as compared to the CT5 and DT5 and DM5 groups, was shown (Table S6). These data indicate a weakening or even absence of the stimulating effect of a five-day-administered gonadotropin on the synthesis of testosterone and its precursors in the testes, especially in MF-treated rats. Moreover, the androstenedione/testosterone ratio did not differ between the DM5, DMT5 and DMG5 groups, which indicates the absence of a significant effect of both LH/hCG-R-agonists on the conversion of androstenedione to testosterone in MF-treated diabetic rats (Table S6).

With a five-day experiment, the intratesticular level of estradiol in the control and diabetic rats did not differ significantly, excluded its decrease in the DG5 group (Table 5). MF treatment reduced estradiol levels as compared with the C5 and D5 groups, but five-day TP3 administration significantly increased it (Table 5). In the DMT5 and DMG5 groups, the estradiol level was 58 and 57% higher than in the corresponding DMT1 and DMG1 groups (Table 4 and Table 5), which indicates estradiol accumulation in the testes of MF-treated rats during five-day LH/hCG-R-agonists administration. In contrast to a single injection of LH/hCG-R-agonists, in MF-treated rats, the stimulating effect of 5-day administration of TP3 on the testosterone/estradiol ratio was not detected, and in the case of hCG it was significantly weakened, which is due to both the accumulation of estradiol and the weakening of testosterone production (Table S6).

### 2.4. Effects of Metformin, LH/hCG-R-Agonists and Their Combination on Gene Expression in the Testes of Control and Diabetic Rats

**Figure 3:**
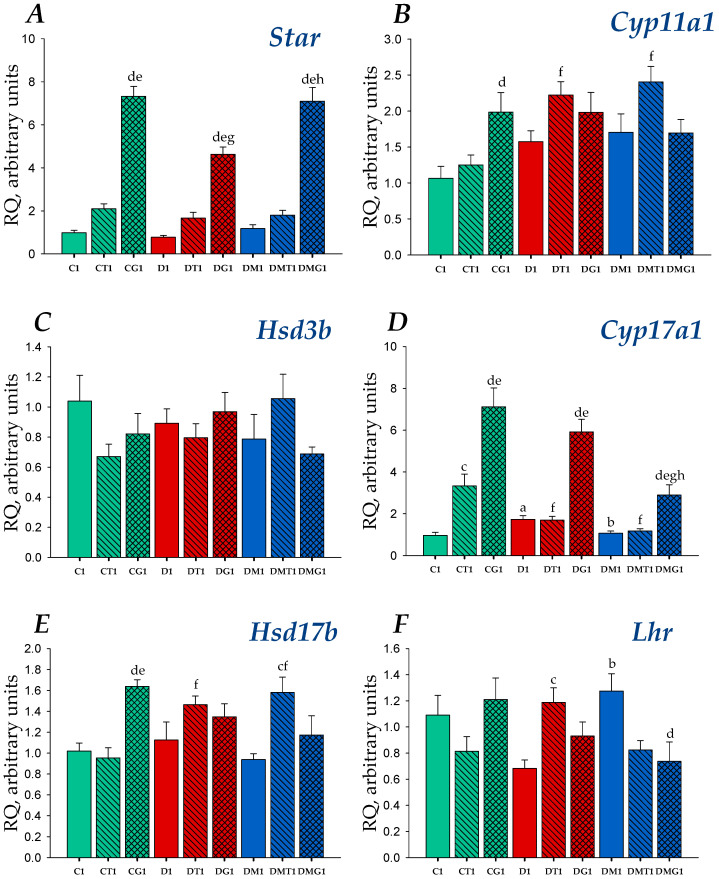

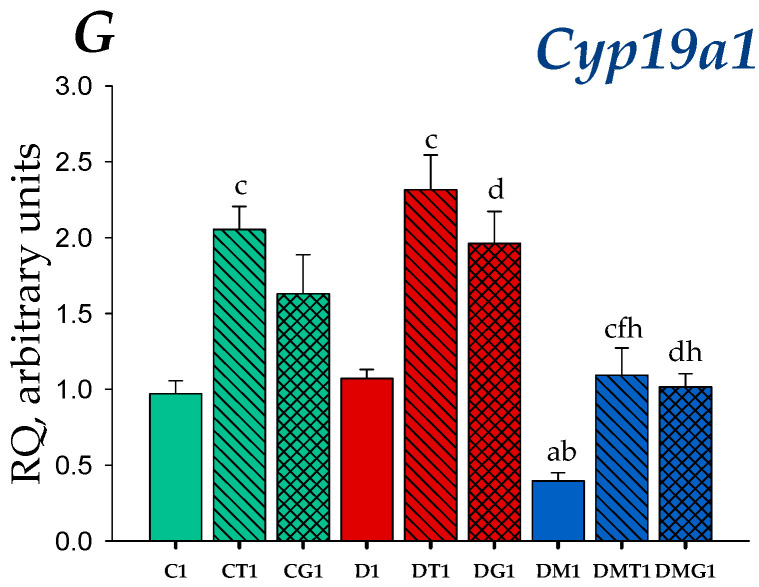
The influence of a single treatment with hCG and TP3 on the expression of testicular genes encoding cholesterol-transporting protein StAR, steroidogenic enzymes and LH/hCG-R in the control and diabetic rats, and the effect of five-week metformin treatment. The duration of MF treatment (120 mg/kg/day) was five weeks. The samples for the assessment of gene expression in the testes were taken 5 h after the injection of LH/hCG-R-agonists. The expression of the following genes is shown in the Figure: *Star*, encoding cholesterol-transporting protein StAR (**A**); *Cyp11a1* (**B**), *Hsd3b* (**C**), *Cyp17a1* (**D**) and *Hsd17b* (**E**), encoding four steroidogenic enzymes cytochrome P450_scc_, 3β-hydroxysteroid dehydrogenase, cytochrome P450 17A1/steroid 17α-monooxigenase and 17β-hydroxysteroid dehydrogenase that catalyze the synthesis of pregnenolone, progesterone, 17-hydroxyprogesterone, androstenedione and testosterone; *LhR,* encoding LH/hCG receptor (**F**); and *Cyp19a1* (**G**), encoding aromatase that catalyzes the synthesis of estradiol. ^a^—the difference between the C1 vs. D1 or DM1, ^b^—the difference between the D1 vs. DM1; ^c^—the difference between the C1 vs. CT1, D1 vs. DT1 and DM1 vs. DMT1; ^d^—the difference between the C1 vs. CG1, D1 vs. DG1 and DM1 vs. DMG1; ^e^—the difference between the CT1 vs. CG1, DT1 vs. DG1 and DMT1 vs. DMG1; ^f^—the difference between the CT1 vs. DT1 or DMT1; ^g^—the difference between the CG1 vs. DG1 or DMG1; and ^h^—the difference between the DT1 vs. DMT1 and DG1 vs. DMG1 are significant at *p* < 0.05. The data are presented as the M ± SEM, *n* = 5.

**Figure 4:**
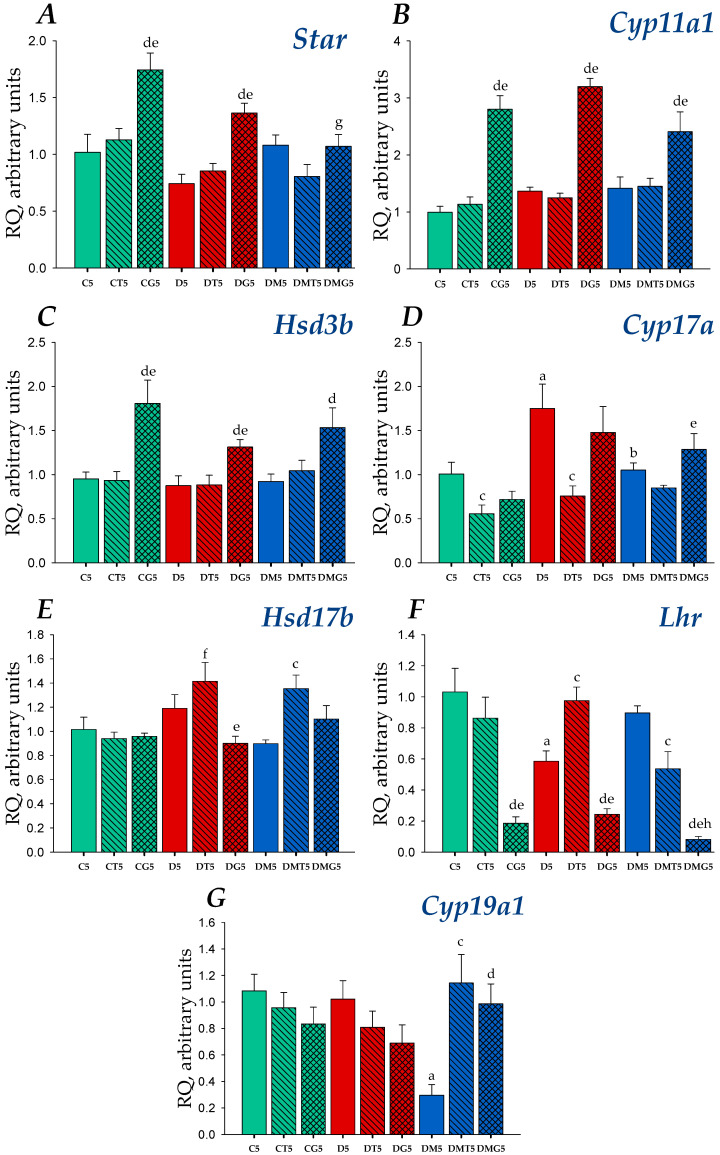
The influence of five-day treatment with hCG and TP3 on the expression of testicular genes encoding cholesterol-transporting protein StAR, steroidogenic enzymes and LH/hCG-R in the control and diabetic rats, and the effect of five-week metformin treatment. The duration of MF treatment (120 mg/kg/day) was five weeks. The samples for the assessment of gene expression in the testes were taken 5 h after the last injection of LH/hCG-R-agonists. The expression of the following genes is shown in the Figure: *Star*, encoding cholesterol-transporting protein StAR (**A**); *Cyp11a1* (**B**), *Hsd3b* (**C**), *Cyp17a1* (**D**) and *Hsd17b* (**E**), encoding four steroidogenic enzymes cytochrome P450_scc_, 3β-hydroxysteroid dehydrogenase, cytochrome P450 17A1/steroid 17α-monooxigenase and 17β-hydroxysteroid dehydrogenase that catalyze the synthesis of pregnenolone, progesterone, 17-hydroxyprogesterone, androstenedione and testosterone; *LhR,* encoding LH/hCG receptor (**F**); and *Cyp19a1* (**G**), encoding aromatase that catalyzes the synthesis of estradiol. ^a^—the difference between the C5 vs. D5 or DM5, ^b^—the difference between the D5 vs. DM5; ^c^—the difference between the C5 vs. CT5, D5 vs. DT5 and DM5 vs. DMT5; ^d^—the difference between the C5 vs. CG5, D5 vs. DG5 and DM5 vs. DMG5; ^e^—the difference between the CT5 vs. CG5, DT5 vs. DG5 and DMT5 vs. DMG5; ^f^—the difference between the CT5 vs. DT5; ^g^—the difference between the CG5 vs. DMG5; and ^h^—the difference between the DG5 vs. DMG5 are significant at *p* < 0.05. The data are presented as the M ± SEM, *n* = 5.

In the rat testes, the expression of the genes encoding LH/hCG-R (gene *Lhr*), cholesterol-transporting protein StAR (gene *StAR*) and five steroidogenic enzymes catalyzing the synthesis of pregnenolone (cytochrome P450_scc_, CYP11A1, gene *Cyp11a1*), progesterone (3β-hydroxysteroid dehydrogenase, 3β-HSD, gene *Hsd3b*), 17-hydroxyprogesterone, androstenedione (cytochrome P450 17A1/steroid 17α-monooxygenase, CYP17A1, gene *Cyp17a1*), testosterone (17β-hydroxysteroid dehydrogenase, 17β-HSD, gene *Hsd17b*) and estradiol (aromatase, gene *Cyp19a1*) was studied. In the diabetic testes, the expression of the gene *Cyp17a1* was increased, while MF treatment reduced and normalized the expression of this gene (Figure 3 and Figure 4). Both untreated and MF-treated diabetic rats tended to increase the expression of the *Cyp11a1* gene (Figure 3 and Figure 4). Diabetic rats had a lowered expression of the *Lhr* gene. In MF-treated rats, the *Lhr* expression was restored, which indicates MF-mediated normalization of LH/hCG-R expression in HFD/STZ-induced T2DM (Figure 3 and Figure 4).

With a single injection, in control rats, TP3 and hCG stimulated the expression of the *Cyp17a1* gene, and gonadotropin was much more effective (Figure 3). hCG also increased the expression of the genes encoding StAR, CYP11A1 and 17β-HSD (Figure 3). TP3 doubled the expression of the *StAR* gene, but the difference from the C1 group was not significant (*p* = 0.058) (Figure 3). In the DG1 and DMG1 groups, hCG increased the *StAR* and *Cyp17a1* expression, while TP3 significantly increased only the *Hsd17b* expression in the DMT1 group (Figure 3). The stimulating effect of gonadotropin on the expression of the *StAR* gene in the MF-treated group was more pronounced than in T2DM and was similar to that in the control animals (Figure 3). In the testes of the DT1 and DMT1 rats, the expression of the *Cyp11a* and *Hsd17b* genes was higher than in the CT1 rats, while the *Cyp17a1* expression was decreased as compared with the CT1 group (Figure 3). In the DT1 group, the *Lhr* expression was increased in comparison with untreated diabetic rats and showed a tendency to increase the expression of this gene compared to that in the CT1 group (*p* = 0.052) (Figure 3). In the DMG1 group, the *Lhr* expression was decreased in comparison with the DM1 group, but there were no differences with the control animals (Figure 3). Thus, the changes of gene expression for animals treated once with TP3 or hCG differed significantly, and in the case of gonadotropin, the changes in the expression of steroidogenic genes and the *Lhr* gene were more pronounced.

After five-day administration of LH/hCG-R-agonists, in control and diabetic rats, hCG increased the expression of the genes encoding StAR, CYP11A1 and 3β-HSD, which catalyze earlier stages of steroidogenesis (Figure 4). Unlike a single injection, there was no stimulation of the *Cyp17a1* expression, and the hCG-induced increase in the *StAR* expression was many times less (Figure 3 and Figure 4). Treatment with hCG led to a significant decrease in the *Lhr* expression as compared to the C5 and D5 groups (Figure 4). TP3 induced a decrease in the expression of *Cyp17a1* gene, and in the DT5 group restored the *Lhr* gene expression reduced in T2DM (Figure 4). In other cases, TP3 had a little effect on the expression of the *Lhr* gene and other steroidogenic genes (Figure 4). In MF-treated rats, hCG increased the *Cyp11a1* and *Hsd3b* expression and significantly reduced the *Lhr* expression, while TP3 increased the *Hsd17b* expression and, to a small extent, reduced the *Lhr* expression (Figure 4). It should be noted that, in comparison with the DM5 group, hCG reduced the expression of the *Lhr* gene by 11 times, while TP3 only by 40% (Figure 4).

In diabetic rats, the testicular expression of the *Cyp19a1* gene encoding aromatase did not differ from that in control animals (Figure 3 and Figure 4). A single injection of TP3 increased the *Cyp19a1* expression in both control and diabetic rats, while hCG increased significantly the expression of aromatase gene only in the DG1 group (Figure 3). The MF treatment led to a significant decrease in the *Cyp19a1* expression, and both TP3 and hCG increased it to control level (Figure 3). In the case of a five-day treatment, TP3 and hCG did not significantly affect the expression of the *Cyp19a1* gene in the testes of control and diabetic rats, while in MF-treated animals, they significantly increased it (Figure 4), as in the case of a one-day administration of the drugs (Figure 3).

### 2.5. The Effects of Metformin, LH/hCG-R-Agonists and Their Combination on the Sperm Parameters in Control and Diabetic Rats

**Figure 5:**
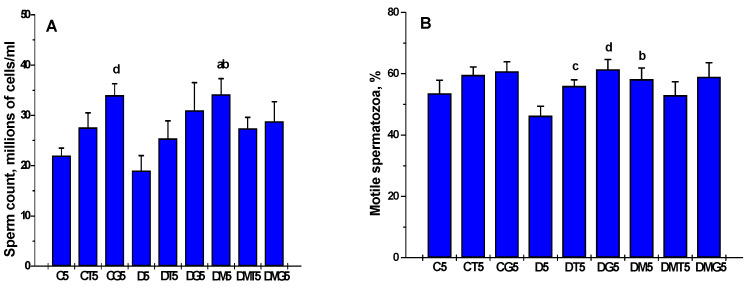

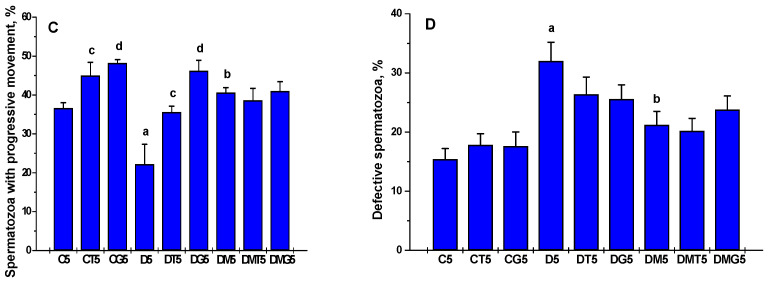
The effects of five-week metformin treatment, five-day treatment with LH/hCG-R-agonists and their combination on the sperm count (**A**), proportion of motile sperm (**B**), proportion of sperm with progressive movement (**C**), and proportion of sperm with tail and head morphological defects (**D**) in the control and diabetic rats. ^a^—the difference between the C5 vs. D5 or DM5, ^b^—the difference between the D5 vs. DM5; ^c^—the difference between the C5 vs. CT5 and D5 vs. DT5; and ^d^—the difference between the C5 vs. CG5 and D5 vs. DG5 are significant at *p* < 0.05. The data are presented as the M ± SEM, *n* = 5.

In diabetic rats, the proportion of epididymal spermatozoa with progressive movement was decreased, and the proportion of spermatozoa with tail and head defects was increased, as compared to control animals (Figure 5). Five-week MF treatment led to an increase in the proportion of motile spermatozoa and progressively moving spermatozoa and to a decrease in their defective forms in comparison to untreated diabetic rats (Figure 5). Along with this, in the DM5 group, the total number of spermatozoa exceeded that in the control group (Figure 5). Five-day treatment of control rats with hCG increased the sperm count and the proportion of spermatozoa with progressive movement, while TP3 increased the proportion of progressively moving spermatozoa (Figure 5). In the case of TP3 treatment, there was a trend towards an increase in the total sperm count, but the difference from the control was not significant (Figure 5).

In diabetic rats, both hCG and TP3 increased the proportion of motile spermatozoa and progressively motile spermatozoa, and hCG was more effective than TP3 (Figure 5). The sperm count tended to increase, but the differences from the control were not significant (Figure 5). In the MF-treated groups, the stimulating effects of TP3 and hCG on sperm motility were not detected (Figure 5). Furthermore, unlike the DM5 group, the total sperm count in the DMT5 and DMG5 groups did not differ significantly from the C5 and D5 groups (Figure 5). All these data indicate that the five-day administration of hCG and TP3 to MF-treated diabetic rats was not able to improve the restorative effect on spermatogenesis, which was achieved with the use of MF monotherapy.

### 2.6. The Effects of Metformin, LH/hCG-R-Agonists and Their Combination on Morphology of the Seminiferous Tubules in Control and Diabetic Rats

**Figure 6:**
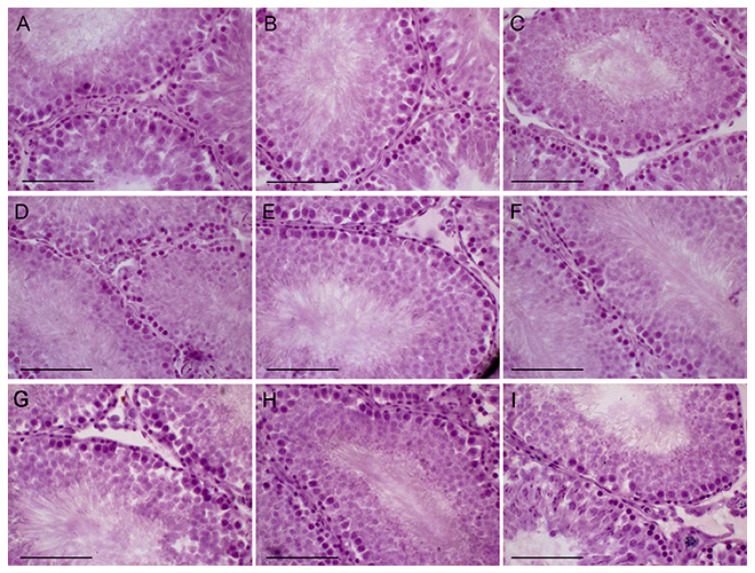
The histochemical analysis of the seminiferous tubules in the testes of control and diabetic rats, and the effect of long-term metformin treatment and five-day treatment with LH/hCG-R-agonists. (**A**)—C5, (**B**)—CT5, (**C**)—CG5, (**D**)—D5, (**E**)—DT5, (**F**)—DG5, (**G**)—DM5, (**H**)—DMT5, (**I**)—DMG5. The histology of the rat testes was evaluated using the staining of the testis section with Mahler’s hematoxylin. Scale bars, 100 µm.

The histological analysis of the testis sections showed that in diabetic rats the thickness of the seminiferous tubule epithelium and the number of spermatogonia and pachytene spermatocytes were reduced and restored when rats were treated with MF (Figure 6, Table 6). In control animals, five-day treatment with TP3 and hCG did not affect the thickness of the epithelium, but significantly increased the number of spermatogonia and pachytene spermatocytes (Table 6). In T2DM, both LH/hCG-R-agonists increased the estimated sperm indices (Figure 6, Table 6). In general, sperm indices in the DMT5 and DMG5 groups were similar to those in the DM5 group and did not differ from the DT5 and DG5 groups (Figure 6, Table 6). An exception was a slight increase in the thickness of the epithelium in the DMT5 group (Table 6).

### 2.7. Immunohistochemical Analysis of LH/hCG-R Distribution in the Seminiferous Tubules of Control and Diabetic Rats, and the Effect of Five-Week Metformin Treatment, Five-Day Treatment with LH/hCG-R-Agonists and Their Combination

**Figure 7:**
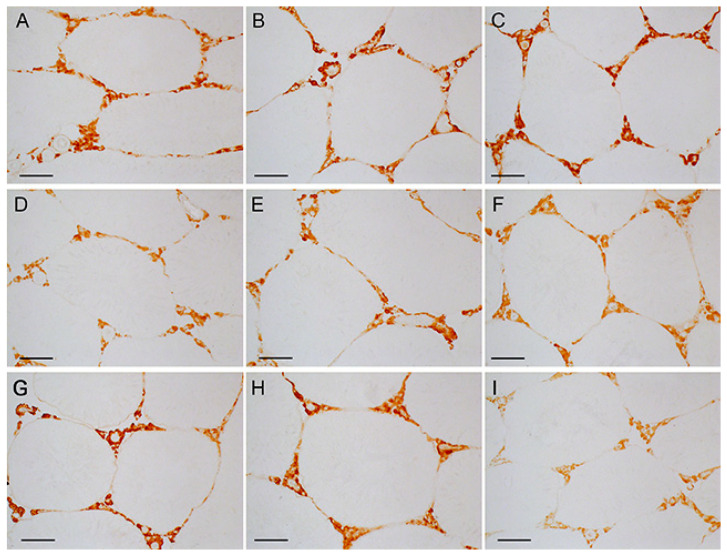
The immunohistochemical analysis of the distribution of LH/hCG-R in the seminiferous Table 5. (**B**)—CT5, (**C**)—CG5, (**D**)—D5, (**E**)—DT5, (**F**)—DG5, (**G**)—DM5, (**H**)—DMT5, (**I**)—DMG5. Scale bars, 100 µm.

**Figure 8:**
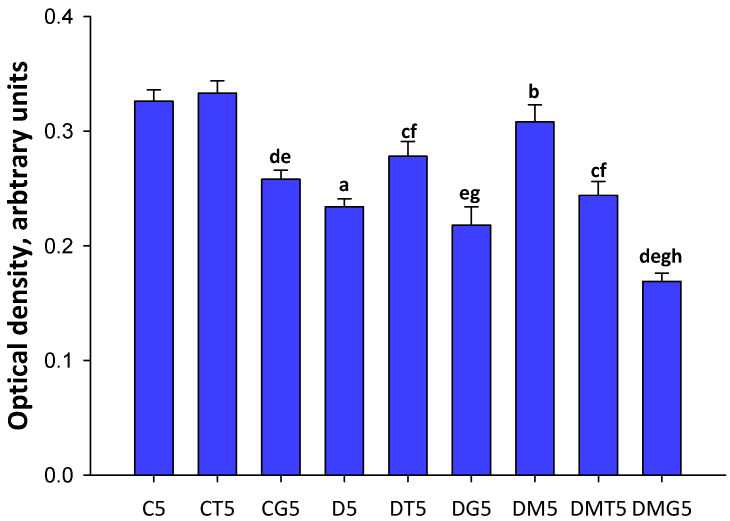
The optical density for LH/hCG-R immunostaining in the testis of control and diabetic rats, and the effect of metformin and LH/hCG-R-agonists treatment. ^a^—the difference between the C5 vs. D5; ^b^—the difference between the D5 vs. DM5; ^c^—the difference between the D5 vs. DT5 and DM5 vs. DMT5; ^d^—the difference between the C5 vs. CG5 and DM5 vs. DMG5; ^e^—the difference between the CT5 vs. CG5, DT5 vs. DG5 and DMT5 vs. DMG5; ^f^—the difference between the CT5 vs. DT5 or DMT5; ^g^—the difference between the CG5 vs. DG5 or DMG5; and ^h^—the difference between the DG5 vs. DMG5 are significant at *p* < 0.05. The data are presented as the M ± SEM, *n* = 5 (10 measurements in each case).

The immunohistochemical content of LH/hCG-R in the testes of diabetic rats was significantly reduced, and the MF treatment restored it (Figure 7 and Figure 8). In control rats, a five-day hCG treatment reduced the LH/hCG-R content, while TP3 did not affect it. In T2DM, the LH/hCG-R content was increased in rats treated with TP3, but not with hCG (Figure 7 and Figure 8). Unexpectedly, but in MF-treated rats, both LH/hCG-R-agonists induced a decrease in the LH/hCG-R content, and in the DMG5 group the LH/hCG-R content was reduced by 2 times in comparison with the C5 and DM5 groups (Figure 7 and Figure 8). It should be emphasized that similar and even more pronounced effects were shown for the *Lhr* expression in the testes of MF-treated animals (Figure 4).

### 2.8. Western Blotting Analysis of α-AMPK and Its Active Thr^172^-Phosphorylated form in the Testes, and the Effect of Metformin and LH/hCG-R-Agonists Treatment

**Figure 9:**
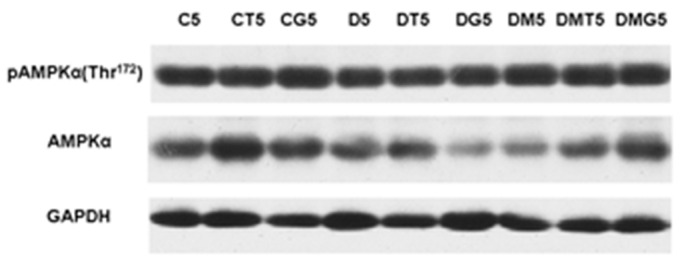
The immunoblots of α-AMPK and Thr^172^-phosphorylated α-AMPK as compared to the reference protein GAPDH in the testes of control and diabetic rats, and the effects of the MF and LH/hCG-R-agonists treatment.

In the testes of diabetic rats, the immunochemical content of α-AMPK tended to decrease, although the differences from the control were not significant (*p* > 0.05). The content of α-AMPK in the DT5 and DG5 groups also showed a tendency to decrease, but in this case the differences with the corresponding control groups were not significant (*p* > 0.05) (Figure 9, Table 7). MF treatment did not change the enzyme content in comparison with the diabetic group, but the α-AMPK expression in the DM5 group was significantly lower than in the control (Figure 9, Table 7). To assess the degree of enzyme activation, the ratio of Thr^172^-phosphorylated (active) α-AMPK to the total α-AMPK is usually used. In our case, a significant increase in this ratio was observed in the DM5 group (Figure 9, Table 7). However, in this group, the content of total α-AMPK was reduced, which negates the significance of an increase in the ratio of the phosphorylated and total α-AMPK. In addition, the evaluation of the ratios of Thr^172^-phosphorylated α-AMPKA and the reference protein GAPDH did not reveal significant differences between the studied groups (Table 7). These data indicate that there is no significant effect of MF treatment on testicular AMPK activity and do not support the possibility of MF- or LH/hCG-R-agonist-mediated enzyme hyperactivation.

## 3. Discussion

Long-term T2DM in male patients often lead to dysfunctions of the hypothalamic-pituitary-gonadal axis, a decrease in testosterone production and a decrease in fertility, which has been demonstrated both in human [51,52,53,54,55,56,57,58,59,60] and on experimental models of these metabolic diseases in rodents [43,61,62,63,64,65]. The main causes of functional changes in the male reproductive system in T2DM are postprandial hyperglycemia, insulin and leptin resistance, inflammation, elevated production of the reactive oxygen and nitrogen species, oxidative stress and lipotoxicity [65,66,67,68]. To study the effect of diabetic pathology on the gonadal axis, we used a model of T2DM in male rats, induced by HFD and low-dose STZ. This model for a number of metabolic and hormonal parameters is most similar to moderate or severe T2DM in human [69], which makes this model the most suitable for studying T2DM-induced reproductive dysfunctions.

We have shown that in diabetic rats, in addition to metabolic changes, testosterone levels in the blood and testes and the testosterone/estradiol ratio were significantly decreased (Table 2, Table 3, Table 4 and Table 5 and Tables S5 and S6; Figure 1 and Figure 2). Along with this, the proportion of spermatozoa with progressive movement was reduced (Figure 5). A histochemical analysis of the diabetic testes showed a decrease in the thickness of the seminiferous epithelium and the number of spermatogonia and pachytene spermatocytes (Figure 6; Table 6). Earlier, we and other authors have also demonstrated a decrease in the level of androgens, and an impaired spermatogenesis in rats with different models of T2DM [43,63,64,65]. In the testes of diabetic rats, the structural disruption and atrophy of the seminiferous tubules, the reduced number of spermatogenic cells and their degeneration and disorganization and the disappearance of spermatids in the lumen of the seminiferous tubules were shown [43,63,65].

In the diabetic testes, gene expression and LH/hCG-R content were reduced (Figure 3, Figure 4, Figure 7 and Figure 8), which is consistent with previously obtained data on a decrease in the expression of LH/hCG-R in the testicular cells of diabetic animals with T2DM and T1DM [38,43,64,70]. A decrease in LH/hCG-R expression should result in a weakening of the steroidogenic response of Leydig cells to endogenous gonadotropins and, we believe, is one of the key causes of androgen deficiency in T2DM male rats. Indeed, in this study we have shown that the stimulating effects of single-dose hCG on testosterone production in T2DM rats were less pronounced than in the control animals (Table 2, Figure 1), and this is in agreement with our early data on the weakening of the gonadotropin steroidogenic effect in T2DM and T1DM rats with reduced LH/hCG-R content in the testes [38,43].

With a single injection of LH/hCG-R-agonists, in both control and diabetic rats the stimulating effect of hCG on testosterone production was higher than that of TP3, especially 1 and 3 h after administration (Table 2, Figure 1), which is due to the higher affinity of hCG to the receptor and the high efficiency of hCG-induced activation of cAMP-dependent signaling responsible for triggering testicular steroidogenesis. This is not surprising since an orthosteric agonist is generally more effective in activating the hormonal receptors than an allosteric agonist [38,71]. However, starting from the second day of LH/hCG-R-agonists treatment, in both control and diabetic rats the steroidogenic effect of hCG weakened and did not significantly differ from the corresponding effect of TP3, which changed to a small extent (Table 3 and Table 5, Figure 2). We believe that this is largely due to a decrease in the number of LH/hCG-R in the testes of hCG-treated rats.

We showed a decrease in the *Lhr* expression and LH/hCG-R content in the testes of control and diabetic rats after five-day treatment with hCG (Figure 4, Figure 7 and Figure 8). It should be noted that the five-day treatment with TP3 did not significantly affect the LH/hCG-R expression, and, as a result, the TP3 effect on the testosterone level did not change significantly during all days of treatment (Figure 2). The other authors showed that hyperactivation of LH/hCG-R with chronic administration of gonadotropin leads to desensitization and down-regulation of the receptor [72,73,74,75,76], and in addition to this, the increased testosterone levels through a negative feedback mechanism suppress the expression of the *Lhr* gene [74]. According to our early data, treatment of rats with TP4/2, another allosteric LH/hCG-R-agonist, had a little effect on the *Lhr* expression [38]. Taken together, these data indicate that long-term treatment with allosteric agonists, contrast to gonadotropins, provide a normal level of LH/hCG-R expression. This may be due to the peculiarities of the regulatory effects of allosteric LH/hCG-R-agonists, namely, with the selective activation of the cAMP signaling cascade and with a more moderate stimulating effect on testosterone production.

It should be noted that no decrease in the expression of genes encoding the main steroidogenic enzymes was found in the testes of diabetic rats (Table 4 and Table 5). Only a tendency towards weakening of the expression of gene for cholesterol-transporting protein StAR, which is responsible for the first, rate-limiting stage of steroidogenesis, was shown (Figure 3 and Figure 4). Other authors also demonstrated a decrease in the expression of the *StAR* gene in the testes of rats with T2DM and HFD-induced obesity [64,77,78,79,80]. Despite a significant decrease in testosterone production in T2DM, we showed an increase in the gene expression for CYP17A1 cytochrome responsible for the synthesis of 17-OH-progesterone and androstenedione (Figure 3 and Figure 4). Meanwhile, the other authors demonstrated both the absence of changes in the expression of this gene [78] and a decrease in its expression [80]. It can be assumed that these differences are due to the severity of diabetic models and its duration. There is evidence that insulin-induced increase in the expression of steroidogenic enzymes, reduced in severe, insulin-deficient STZ diabetes, does not lead to the normalization of steroidogenesis [81]. This is due to the fact that, under the diabetic conditions, the processing of testicular proteins is impaired, as a result of the increased oxidative stress and stress of the endoplasmic reticulum. As a consequence, the normalization of gene expression of the steroidogenic enzyme is often not associated with the restoration of its activity. A clearly pronounced correlation between the gene expression and activity is shown only for the StAR protein [45,82].

With a single injection, LH/hCG-R-agonists differently influenced the expression of steroidogenic genes and the ratios of steroid hormones. In T2DM, the hCG stimulating effect on the *StAR* expression was decreased, while its stimulating effects on the *Cyp11a1* and *Cyp17a1* expression changed to a lesser extent (Figure 3). In the diabetic testes, in contrast to control, the stimulating effect of TP3 on the *Cyp17a1* expression was not detected, while its stimulating effect on the *Cyp11a1* expression was appeared (Figure 3). With five-day treatment, the hCG stimulating effect on the *Cyp11a1* expression was increased, while the corresponding effect on the *Cyp17a1* expression disappeared (Figure 4). Thereby, with T2DM and with an increase in the duration of LH/hCG-R-agonists treatment, the *StAR* expression was decreased, and there were also multidirectional changes in the expression of steroidogenic enzymes that can compensate for dysfunctions of testicular steroidogenesis.

MF, which is widely used for T2DM treatment, not only improves metabolic status and insulin sensitivity, but also restores functions of the cardiovascular, urinary, nervous and other systems impaired in diabetic conditions [17,83,84,85,86,87,88]. MF also has a positive effect on the reproductive system and fertility. There is strong evidence for the therapeutic potential of MF to restore the functions of the female reproductive system in T2DM, obesity, and polycystic ovary syndrome [8,89,90,91,92,93]. Much less is known about the restorative effect of MF on the male reproduction in T2DM and metabolic syndrome, which is largely due to the limited number of clinical studies. A number of researchers point to an improving effect of MF on spermatogenesis and androgenic status in male patients with metabolic diseases [6,94,95,96]. Morgante and coauthors showed that six-month MF therapy of male patients with metabolic syndrome restored a number of spermatozoa, their motility and morphology, normalized the testosterone levels and, thereby, improved fertility [95]. However, it should be noted that a number of authors have not shown a significant restorative effect of MF therapy on spermatogenesis and androgenic status in T2DM and obesity [97,98], or indicate the need for combined use of MF with other drugs [99].

Most studies carried out on experimental models of diabetes and obesity support a significant improving effect of MF on spermatogenesis and testicular steroidogenesis [7,19,50,70,78,100,101,102,103,104]. Moreover, both monotherapy with MF and its co-administration with pioglitazone [101] and natural antioxidants [78,105,106] were effective in the restoration of reproductive functions. In the present study, we found that in male rats with HFD/STZ-induced T2DM, a five-week treatment with MF partially restored the testosterone levels in the blood and testes and normalized the ratios between testosterone and its precursors (Table 2, Table 3, Table 4 and Table 5 and Tables S5 and S6; Figure 1 and Figure 2), improved morphology of the seminiferous tubules, restored the number of spermatogonia and pachytene spermatocytes in them (Table 6), increased the total number of epididymal spermatozoa, their motility and the proportion of spermatozoa with progressive movement, and also reduced the number of their defective forms (Figure 5). Moreover, the sperm count in MF-treated diabetic rats exceeded those in the control group (Figure 5).

We have shown for the first time a pronounced improving effect of MF therapy on the steroidogenic effect of both LH/hCG-R-agonists after their single injection to diabetic rats. Five hours after TP3 and hCG administration, the blood and testicular levels of testosterone and the ratio for testosterone and its precursors in the testes were comparable to that in control rats treated with the same agonists (Table 2, Table 4 and Tables S5 and S6). hCG-stimulated *StAR* expression in the DMG1 group exceeded that in the DG1 group and was comparable to the *StAR* expression in the CG1 group (Figure 3). The expression of the *StAR* gene in the DMT1 group was similar to that in the CT1 group (Figure 3). Since the StAR plays a key role in triggering steroidogenesis [82,107], MF-mediated restoration of the stimulating effects of LH/hCG-R-agonists on the *StAR* expression may be an important factor in enhancing their steroidogenic activity.

MF normalized the *Lhr* gene expression and the LH/hCG-R content in the diabetic testes (Figure 3, Figure 4, Figure 7 and Figure 8). From our point of view, this makes a significant contribution to improving the sensitivity of Leydig cells to LH/hCG-R-agonists, which was demonstrated in our experiments with a single administration of hCG and TP3 to MF-treated rats (Table 2, Figure 1). There is evidence that some other antidiabetic drugs are able to increase the LH/hCG-R expression, as shown for carvacrol, a potent antioxidant that increases the expression of gonadotropin receptors in the Sertoli cells of diabetic rats [108].

Based on our data discussed above, we expected that MF would enhance the effects of LH/hCG-R-agonists on testosterone production and spermatogenesis after long-term, five-day, administration. However, in this case, improving effect of MF therapy on TP3 and hCG effects was not observed (Table 3 and Table 5, Figure 2). In diabetic rats without MF treatment, the five-day administration of TP3 and, to a greater extent, hCG normalized the thickness of the epithelium of the seminiferous tubules, the number of spermatogonia and pachytene spermatocytes (Table 6), and, in addition, increased the number of epididymal spermatozoa and the proportion of their motile forms (Figure 5). At the same time, in MF-treated rats, the improving effect of TP3 and hCG on spermatogenesis indices was disappeared, and in some cases, there was a tendency for their deterioration (Figure 5, Table 6). As an example, in contrast to the DM5 group, where the number of spermatozoa exceeded the control values, this was not shown in the DMT5 and DMG5 groups, and the proportion of motile spermatozoa in the DMT5 group was similar to that in the diabetic group (Figure 5).

The data obtained indicate that under the conditions of MF-induced normalization of steroidogenesis and spermatogenesis, further improvement of the reproductive system in diabetic rats during their chronic treatment with LH/hCG-R-agonists becomes hardly achievable. Moreover, an excessive increase in intratesticular testosterone can negatively affect spermatogenesis and impair sperm fertility [109,110]. There are many studies that long-term administration of testosterone drugs damages sperm and can cause infertility [111,112,113,114]. Therefore, long-term hormonal activation of LH/hCG-R should trigger mechanisms that prevent “excessive” activation of testicular steroidogenesis. Accordingly, we have shown that in rats treated with MF, one of such mechanisms is a pronounced decrease in testicular expression of the *Lhr* gene in response to a five-day administration of LH/hCG-R-agonists. In this case, the *Lhr* expression upon administration of TP3 and hCG was decreased by 40 and 91% compared to the DM5 group (Figure 4). Meanwhile, in the testes of diabetic rats without MF treatment, five-day administration of hCG reduced the expression of the *Lhr* gene by only 58%, while TP3 increased it by 66% (Figure 4). Using immunohistochemical approach, we showed that the testicular content of LH/hCG-R in the DMT5 and DMG5 groups was decreased by 21 and 45% in comparison with the DM5 group, and in the DMG5 group it was significantly lower than in the D5, DT5 and DG5 groups (Figure 7 and Figure 8). We believe that a long-term increase in testosterone level as a negative regulator of the gonadal axis [115,116] may play a decisive role in the decrease in LH/hCG-R expression in the DMT5 and DMG5 groups. It cannot be ruled out that, in addition to stimulation of steroidogenic cAMP-dependent pathways, long-term activation by LH/hCG-R-agonists provokes down-regulation of LH/hCG-R, activating G protein-coupled receptor-specific kinases and β-arrestins, which triggers the intracellular cascades that negatively regulate the *Lhr* expression [117,118,119].

It should be noted, however, that in control rats, the same as animals of the DM5 group, the five-day administration of hCG (but not TP3) also significantly reduced the expression of the *Lhr* gene (by 82%) (Figure 4), but in this case, the steroidogenic effect of hCG was maintained, although it was reduced as compared to the first day of treatment (Table 3). Accordingly, in addition to reducing the LH/hCG-R expression, some other mechanisms may be activated in MF-treated rats, mediating an unexpected suppression of the steroidogenic effects of LH/hCG-R-agonists with long-term administration. In the testes, these mechanisms may include the changes in the expression and activity of aromatase, which catalyzes the conversion of androgens to estradiol, a functional testosterone antagonist, and MF-induced hyperactivation of AMPK, an energy sensor of cell.

In obesity and hyperinsulinemia typical for T2DM, the activity of aromatase is enhanced, which leads to a decrease in the testosterone/estrogens ratio and impairs male fertility [120,121]. MF has a direct inhibitory effect on aromatase expression in granulosa luteal cells [122,123]. We showed that in the testes of diabetic rats, MF treatment significantly suppressed the expression of the *Cyp19a1* (aromatase) gene (Figure 3 and Figure 4) and also decreased the estradiol levels in comparison to control and diabetic rats (Table 4 and Table 5) and normalized the testosterone/estrogen ratio (Tables S5 and S6), which we believe is a consequence of weakening the aromatase-catalyzed conversion of androstenedione to estrogens. In control and diabetic rats, a single, but not five-day, administration of LH/hCG-R-agonists increased the *Cyp19a1* expression (Figure 3 and Figure 4). In the MF-treated groups, LH/hCG-R-agonists did not increase the *Cyp19a1* expression above its control level, although they did increase it compared to the DM1 or DM5 groups (Figure 3 and Figure 4). It seems to us that it is more correct to consider the LH/hCG-R-agonists effect as the cancellation of MF inhibitory influence on the *Cyp19a1* expression. With regard to estradiol production in MF-treated rats, the effects of single and five-day administration of LH/hCG-R-agonists differed. With a single injection, the estradiol level reduced in the DM1 group tended to decrease even more (Table 4), while with five-day treatment, this level in LH/hCG-R-agonist-treated groups was increased, and in the case of TP3, the difference with the DM5 group was significant (Table 5). In the DMT5 and DMG5 groups, the testosterone/estradiol ratio was 56 and 70% lower than in the DMT1 and DMG1 groups. Addition to this, in the DMT5 and DMG5 groups, this ratio was also reduced in comparison with the CT5 and CG5 groups (by 36 and 48%) and with the DT5 and DG5 groups (by 33 and 34%), respectively (Tables S5 and S6). Thereby, a single injection of LH/hCG-R-agonists to MF-treated rats increased the testosterone/estradiol ratio, making a significant contribution to the enhancement of androgen-regulated processes in the DMT1 and DMG1 rats, while the five-day treatment led to the opposite effect.

Hyperactivation of AMPK in the testes and ovaries can lead to impaired production of steroid hormones [124,125,126,127,128]. It was shown that an increase in AMPK activity in granulosa cells of broiler chickens leads to a decrease in the expression of genes encoding StAR, cytochrome CYP11A1 and dehydrogenase 3beta-HSD, causing a decrease in progesterone synthesis [126]. Hyperactivation of AMPK in luteal cells suppresses steroidogenesis, and an important role in this is played not by the inhibition of steroidogenic proteins, but by the inhibition of the hormone-sensitive lipase HSL. This enzyme hydrolyzes cholesterol esters and makes cholesterol available for transport into the mitochondria [128]. Since MF is one of the activators of AMPK, we assumed that MF treatment can lead to AMPK hyperactivation, which, in turn, is able to inhibit the steroidogenic effect of LH/hCG-R-agonists. However, the immunoblotting data obtained by us indicate that, despite the increase in the ratio of active Thr^172^-phosphorylated α-AMPK and total α-AMPK in the testes of MF-treated rats in comparison with the control rats, the content of Thr^172^-phosphorylated form, assessed by its ratio with the reference protein GAPDH, did not change significantly (Figure 9, Table 7). Thus, there is no reason to believe that in our case MF treatment leads to hyperactivation of testicular AMPK. In addition, five-day treatment with LH/hCG-R-agonists did not significantly affect the content and activity of AMPK, and, moreover, in the DMG5 group, there was a hCG-induced decrease in the ratio of Thr^172^-phosphorylated and total AMPK in comparison with the DM5 group (Figure 9, Table 7). Thus, the dose of MF and the duration of MF therapy we have chosen do not cause AMPK hyperactivation in the testes of diabetic rats and are optimal for restoring metabolism and steroidogenesis in testicular cells. Accordingly, the assumption about the contribution of AMPK hyperactivation to the inhibition of steroidogenic effects of long-term administered LH/hCG-R-agonists in T2DM is not supported by our data.

Of interest are our data that the steroidogenic effect of long-term administration of TP3 changes weakly in T2DM. This may be due to the fact that allosteric agonists to a lesser extent, in comparison with hCG, affect the expression of LH/hCG-R. However, it cannot be ruled out that this is due to the peculiarities of the interaction of TP3 with LH/hCG-R. First, low-molecular-weight allosteric LH/hCG-R-agonists interact with the transmembrane domain of LH/hCG-R, which undergoes fewer changes under the conditions of prolonged hyperglycemia in comparison with the ectodomain to which hCG binds. Second, as shown for the Org43553, another allosteric LH/hCG-R-agonist, such agonists are able to penetrate into the cell and interact there with immature forms of the receptor, facilitating their translocation to the membrane [129]. Based on this, it can be assumed that the steroidogenic effects of TP3 may not be so sensitive to inactivating modifications of LH/hCG-R under hyperglycemia and oxidative stress, typical for T2DM, which are believed to be capable of disrupting the maturation of LH/hCG-R and its translocation into the plasma membrane. We have previously shown the stability of the steroidogenic effect of TP4/2, another LH/hCG-R-agonist we developed, in male rats with STZ diabetes [38]. These data indicate that the use of thieno[2,3-*d*]-pyrimidine LH/hCG-R-agonists is promising for compensating for androgen deficiency in various types of diabetes. At the same time, the advantages of thieno[2,3-*d*]-pyrimidines may disappear when used together with MF, which in T2DM reduces hyperglycemia and improves protein processing, including, presumably, LH/hCG-R.

## 4. Materials and Methods

### 4.1. The Drugs and Biochemical Reagents

The synthesis of 5-amino-*N-tert*-butyl-2-(methylsulfanyl)-4-(3-(nicotinamido)phenyl)thieno[2,3-*d*] pyrimidine-6-carboxamide (TP3), a low-molecular-weight allosteric LH/hCG-R-agonist, was carried out using the acylation of 5-amino-4-(3-aminophenyl)-*N*-(*tert*-butyl)-2-(methylthio)thieno[2,3-*d*]pyrimidine-6-carboxamide, which was obtained according to the method of Hanssen and Timmers [130], as described earlier (Derkach et al., 2016) (Figure S1). For this, 5-amino-4-(3-aminophenyl)-*N*-(*tert*-butyl)-2-(methylthio) thieno[2,3-*d*]-pyrimidine-6-carboxamide (1.0 equivalent) was dissolved in dry *N*,*N*-dimethylformamide and mixed with nicotinic acid (1.1 equivalents) in the presence of *N*,*N*-diisopropylethylamine (1.2 equivalents). Then, 1-[bis(dimethylamino)methylene]-*1H*-1,2,3-triazolo[4,5-*b*]-pyridinium 3-oxide hexafluorophosphate (1.1 equivalents) was added, and the mixture was stirred at the room temperature for 5 h. After removing the solvent in a vacuum evaporator, the resulting residue was washed with 5% sodium carbonate, then distilled water and then filtered. The obtained product was purified by column chromatography and characterized by high-resolution mass spectrometry and the ^1^H- and ^13^C-NMR. The high-resolution mass spectra (electrospray ionization—time of flight, ESI-TOF) were recorded using a “Bruker micrOTOF” spectrometer (“Bruker”, Karlsruhe, Germany). The NMR spectra were obtained using a “Bruker Avance III 400” spectrometer (400.13 MHz for ^1^H and 100.61 MHz for ^13^C) (“Bruker”, Germany) in DMSO-*d*_6_ and were referenced to residual solvent proton signal (*δ*H = 2.50) and solvent carbon signal (*δ*C = 39.5). The obtained compound TP3 (C_24_H_24_N_6_O_2_S_2_) is yellow solid with the melting temperature 157–159 °C; the ^1^H-NMR spectrum (DMSO-*d*_6_), δ, ppm (*J*, Hz): 1.37 (9H, s, C(CH_3_)_3_); 2.62 (3H, s, SCH_3_); 6.14 (2H, s, NH_2_); 6.98 (1H, s, NH-*t*-Bu); 7.42 (1H, d, *J* = 7.8, 4′-H); 7.57–7.64 (2H, m, 5′-H и 5″-H); 8.04 (1H, d, *J* = 8.2, 2′-H); 8.08 (1H, s, 6′-H); 8.33 (1H, d, *J* = 7.7, 4″-H); 8.78 (1H, d, *J* = 4.8, 6″-H); 9.14 (1H, s, 2″-H), 10.72 (1H, s, 3′-NH); and the ^13^C-NMR spectrum (DMSO-*d*_6_), δ, ppm: (14.3 (SCH_3_); 29.2 (C(CH_3_)_3_); 51.9 (C(CH_3_)_3_); 97.7; 117.8; 120.9; 122.5; 124.0; 124.9; 129.8; 130.8; 136.0; 137.1; 139.6; 144.7; 149.2; 152.8; 162.5; 164.9; 165.2; 167.8; 168.8). According to high-resolution mass spectrometry (ESI-TOF) the molecular weight of the compound was 515.1304 (the calculated molecular weight for [M + Na^+^] was 515.1294) (see the spectrum details in the Figure S1).

Metformin, STZ, hCG, inhibitors of proteases (phenylmethylsulfonyl fluoride, *ortho*-phenanthroline, pepstatin) and the other reagents were obtained from “Sigma-Aldrich” (St. Louis, MO, USA).

### 4.2. The Experimental Animals

The male Wistar rats *Rattus norvegicus* (the age before the start of the experiment was 10 weeks; the body weight was 160–180 g) were obtained from the “Rappolovo” nursery (Leningrad Region, Russia). The animals were housed in plastic cages, five animals in each, with a normal light–dark cycle (12 h/12 h, light on at 9.00 a.m.) and the room temperature (24 ± 2 °C), and had free access to standard laboratory chow pellets and drinking water. The daily standard diet contained 20–25 g of dry food (19% protein, 5% fat, 4% fiber, and 9% ash), which provided 2.95 kcal/g. All experiments were approved by the Bioethics Committee at the Sechenov Institute of Evolutionary Physiology and Biochemistry (St. Petersburg, Russia) (protocol No #3/2020, 18 March 2020) and according to the Declaration of Helsinki, “The Guide for the Care and Use of Laboratory Animals” and the European Communities Council Directive of 1986 (86/609/EEC). All the efforts to minimize animal suffering and reduce the number of experimental animals was made.

### 4.3. The Induction of Type 2 Diabetes in Male Wistar Rats

**Figure 10:**
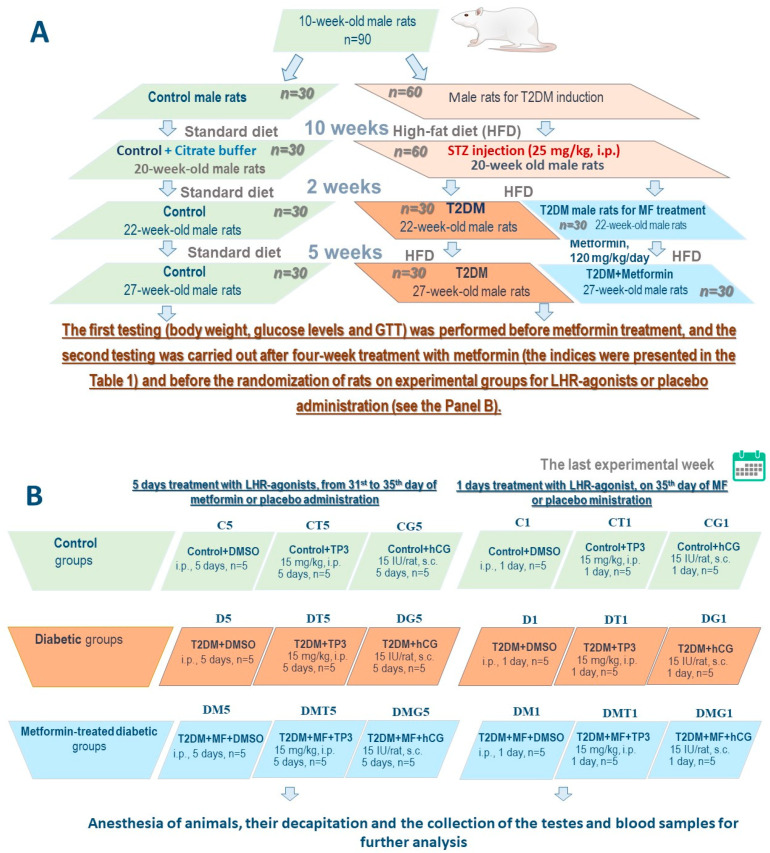
The design of experiments (**A**) and the rat groups (**B**).

T2DM was induced by 10 weeks of HFD followed by a single treatment with low-dose STZ (25 mg/kg, i.p.), as described earlier [69] (Figure 10). Control rats of the same age received a standard diet and instead of STZ were treated with its solvent (0.1 M sodium-citrate buffer, pH 4.5). The HFD included the supplements of 5–7 g of fat mixture containing (*w*/*w*) 52.4% of pork lard, 41.7% of curd, 5% of liver, 0.5% of *L*-methionine, 0.2% of baker’s yeast, and 0.2% of NaCl [69]. After STZ treatment, the rats of the experimental group continued to receive the HFD. Two weeks after STZ injection, among the animals of the experimental group were chosen rats that had obvious signs of T2DM, including the increased body weight, increased content of glycated hemoglobin (HbA1c, above 6%), as well as glucose intolerance according to the results of glucose tolerance test (GTT). In the GTT, 120 min after glucose loading, the glucose levels in diabetic rats were above 8 mM, and the AUC_0–120_ values for the curve “glucose concentration, mM—time, minutes” were above 30% as compared to the average AUC_0–120_ values in the control group.

### 4.4. The Treatment of Diabetic Rats

At the first stage, rats with T2DM were randomized into two groups: untreated diabetic animals (Diabetes, *n* = 30) and diabetic rats treated for five weeks with MF at a daily dose 120 mg/kg (orally using a gavage) (Diabetes + MF, *n* = 30) (Figure 10). Control animals (Control, *n* = 30) instead of MF for five weeks received 0.9% NaCl solution (saline), the solvent of this drug. Four weeks after starting MF treatment, in all the investigated groups the GTT was performed, and the blood samples were taken to measure the levels of glucose, HbA1c, hormones and lipids. At the next stage, all animals were randomized into groups for one-day or five-day treatment with the LH/hCG-R-agonists. Five-day treatment with TP3 (i.p., DMSO, 15 mg/kg/day) and hCG (s.c., saline, 15 IU/rat/day) was carried out from the 31st to the 35th days of MF treatment, while a single injection of LH/hCG-R-agonists at the same doses was carried out on the 35th day of MF treatment (the final day of the experiment). The choice of drug doses was based on preliminary studies of the “drug dose-steroidogenic effect” relationship. The selected doses of TP3 and hCG corresponded to their doses, which caused 60–65% of the maximum steroidogenic effect of the drugs when they were administered once to three-month-old male rats. Groups of rats without administration of LH/hCG-R-agonists received DMSO (150 μL, i.p.). Earlier, we showed that a five-day administration of DMSO to rats, even in a larger volume (200 μL, i.p.), does not affect their metabolic and hormonal parameters and testicular function [38].

Summarizing, nine groups of rats were formed to study the effects of single-dose LH/hCG-R-agonists and other nine groups to study the effects of five-day administration of LH/hCG-R-agonists (in each group *n* = 5). The groups with a single injection of LH/hCG-R-agonists or DMSO included control (C1), control receiving TP3 (CT1) or hCG (CG1), diabetic rats (D1), diabetic rats receiving TP3 (DT1) or hCG (DG1), MF-treated diabetic rats (DM1), MF-treated diabetic rats receiving TP3 (DMT1) or hCG (DMG1). The groups with a five-day treatment with LH/hCG-R-agonists or DMSO included: control (C5), control receiving TP3 (CT5) or hCG (CG5), diabetic rats (D5), diabetic rats receiving TP3 (DT5) or hCG (DG5), MF-treated diabetic rats (DM5), MF-treated diabetic rats receiving TP3 (DMT5) or hCG (DMG5) (Figure 10).

### 4.5. Preparation of Tissue and Blood Samples for Analysis

On the last day of the experiment, the animals were anesthetized by chloral hydrate administration (400 mg/kg b.w., i.p.) and then decapitated. The samples of blood, testes, and epididymis were taken to measure hormone levels in the blood and testes and gene expression in the testes, to estimate sperm parameters and to carry out histochemical analysis of the seminiferous tubules. The testes were weighed and the gonadosomatic index (GSI) was calculated as (testes weight/total body weight) × 100, where the testes weight is the sum of the weight of the right and left testes.

### 4.6. The Determination of Blood Levels of Glucose, HbA1c, Insulin, Leptin, Total Cholesterol and Triglycerides

Glucose levels in the blood obtained from the tail vein were measured using a glucometer (“Life Scan Johnson & Johnson”, Milpitas, CA, USA) and the test-strips “One Touch Ultra” (“LifeScan Inc.”, Malvern, PA, USA). The levels of insulin and leptin in the rat serum were measured using the kits “Rat Insulin ELISA” (“Mercodia AB”, Uppsala, Sweden) and “ELISA for Leptin, Rat” (“Cloud-Clone Corp.”, Houston, TX, USA, Indianapolis, IN, USA), and the content of HbA1c was measured using the “Multi Test HbA1c System kit” (“Polymer Technology Systems, Inc.”, Indianapolis, IN, USA). The concentration of total cholesterol and triglycerides was measured using the express test MulticareIN “Biochemical Systems International S.p.A.” (Arezzo, Italy).

### 4.7. Glucose Tolerance Test

In the Control, Diabetes and Diabetes + MF groups, the GTT was performed using a single injection of glucose (2 g/kg, i.p.) after 12 h of fasting, as described earlier [69]. The blood samples for measuring of glucose, insulin and leptin levels were collected from the tail vein of rats under local anesthesia (Lidocaine, 2%, 2–4 mg/kg b.w.) before (0 min) and 120 min after glucose load. The GTT was performed two days before starting treatment with LH/hCG-R-agonists (four weeks after starting MF treatment).

### 4.8. The Determination of Serum Testosterone Levels and Intratesticular Content of Testosterone, Estradiol and Their Precursors

The serum and intratesticular levels of testosterone were determined using the “Testosterone-ELISA kit” (“Alkor-Bio”, Saint-Petersburg, Russia). The intratesticular content of progesterone, 17-hydroxyprogesterone and estradiol was measured with the kits “Progesterone-EIA”, “17-OH-progesterone-EIA” and “Estradiol-EIA” (“XEMA Co. Ltd.”, Moscow, Russia), and the intratesticular content of androstenedione was estimated using the kit “Androstenedione ELISA” (“DRG Instruments GmbH”, Marburg, Germany).

### 4.9. The RNA Extraction and qRT-PCR Analysis of Testicular Genes

Total RNA was isolated from the rat testes using the “ExtractRNA Reagent” (TRIzol analogue) (“Evrogen”, Moscow, Russia) according to the manufacturer’s instructions. The samples containing 1 μg of RNA were transcribed to cDNA using the random oligodeoxynucleotide primers and the “MMLV RT kit” (“Evrogen”, Moscow, Russia). The amplification was performed using the incubation mixture containing 10 ng of reverse-transcribed product, 0.4 μM of the forward and reverse primers, and the “qPCRmix-HS SYBR + LowROX kit” (“Evrogen”, Moscow, Russia). The amplified signals were detected using the “Applied Biosystems^®^ 7500 Real-Time PCR System” (“Life Technologies, Thermo Fisher Scientific Inc.”, Waltham, MA, USA). The primers that were used to assess the expression of the target genes are presented in the Table 8. The obtained data were calculated using the delta-delta C_t_ method and expressed as fold expression relative to expression in the corresponding control group [131]. The expression of the gene encoding actin B (*Actb*) was used as an endogenous control.

### 4.10. The Analysis of Epididymal Spermatozoa

The number of epididymal spermatozoa and the proportion of their motile and defective forms and spermatozoa with progressive movement were assessed using a Makler’s Counting Chamber, the depth 10 μm (“Sefi Medical Instruments”, Haifa, Israel). Briefly, the left caudal epididymis was carefully incised, and 5 mg of the semen fluid was gently diluted in 195 μL of the “Quinn’s Advantage™ Medium with HEPES” (“In Vitro Fertilization Inc.”, Trumbull, CT, USA) and then incubated for 30 min at 37 °C. Subsequently, 10 μL of diluted semen fluid was added to a Makler’s Counting Chamber. The number of epididymal spermatozoa was counted using a MICMED-5 microscope, the magnification ×400 (“LOMO”, Saint-Petersburg, Russia) and the results were presented as 10^6^ cells/g of the caudal epididymis. Epididymal spermatozoa with progressive movement were calculated as a percentage of the total number of motile spermatozoa, taken as 100%. To study sperm morphology, the sperm smears examined for the defects of head and tail spermatozoa [132]. Each smear was placed on a glass slide, air-dried at the room temperature and then stained with azure and eosin using the “Spermo-Diff-200” kit (“Syntacon”, Saint-Petersburg, Russia). For each slide, 200 spermatozoa were counted with a MICMED-5 microscope (magnification ×1000), and the number of defective forms of epididymal spermatozoa was expressed as a percentage of the total number of spermatozoa, taken as 100%. The epididymal spermatozoa analysis was performed in a blinded fashion with regards to the studied groups.

### 4.11. Preparation of the Testis Sections for Histochemical and Immunohistochemical Analysis

The testes were excised and immediately fixed for 48 h (4 °C) in 4% *para*-formaldehyde solution, washed with 0.9% sodium-phosphate buffer (PBS) and immersed in PBS containing 30% of sucrose. The testes were frozen on dry ice using the Tissue-Tek medium (“Sacura Finetek Europe”, Alphen aan den Rijn, The Netherlands). The series of cross sections of the testes (the thickness is 6 μm) were prepared using a Leica CM-1520 cryostat (“Leica Biosystems”, Nussloch, Germany) and mounted on the SuperFrost/plus glasses (“Menzel”, Braunschweig, Germany). The sections of the seminiferous tubules were mounted on glass in such a way as to obtain the sections from different levels of the testes. The sections from different experimental groups were mounted on one same glass, air-dried overnight, and then used for histochemical and immunohistochemical analysis.

### 4.12. Immunohistochemical Analysis of Intratesticular LH/hCG Receptor

Freshly prepared testes sections were washed with PBS, treated with 0.6% hydrogen peroxide in PBS for 30 min to block endogenous peroxidase activity, washed with PBS for 10 min, and then with PBS containing 0.1% Triton X-100 (PBST) for 20 min. Subsequently, the sections were incubated for 1 h in a blocking solution (5% goat serum in PBST). Incubation with the primary Polyclonal Rabbit anti-Human LHCGR/LHR/LH Receptor Antibody (#LS-C3, “LSBio”, Seattle, WA, USA) was carried out in 1% blocking solution at a dilution of 1:1000 overnight (22 °C). After washing with PBST (40 min), the sections were incubated for 1 h in PBST with the secondary Goat Anti-Rabbit IgG Antibody, Biotinylated (#BA-1000, “Vector Laboratories”, Burlingame, CA, USA) at a dilution of 1:600, then were washed with PBS for 30 min and incubated in a solution of streptavidin-peroxidase (“Sigma-Aldrich”, St. Louis, MO, USA) in PBS at a dilution of 1:600 for 1 h. After washing in PBS, the sections were treated with 0.05% diaminobenzidine (“Sigma-Aldrich”, St. Louis, MO, USA) and 0.03% hydrogen peroxide in PBS. The reaction was stopped by washing with distilled water, after which the sections were placed under a cover glass using glycerol. A specificity of immunohistochemical reaction was checked using a negative control (the samples without the primary or secondary antibodies). The micrographs (20 for each animal) from different levels of the testis and different areas of each section were obtained using a Carl Zeiss Imager A1 microscope, the objective x20 (“Carl Zeiss”, Oberkochen, Germany) using the same optic characteristics for different groups of animals. The optical density was quantified using the Image J NIH Analysis software (“National Institutes of Health”, Bethesda, MD, USA). The optical density of LH/hCG-R-immunopositive material was estimated and expressed in the arbitrary units.

### 4.13. Histochemical Analysis of the Seminiferous Tubules

To study morphology of the seminiferous tubules, the testis sections were treated with 50% ethanol for 15 min, washed with distilled water and stained with Mahler’s hematoxylin according to the standard procedure. Then the sections were washed again in distilled water and placed under a cover glass using glycerol. The seminiferous tubules samples were analyzed using a Carl Zeiss Imager A1 microscope (“Carl Zeiss”, Oberkochen, Germany). With a ×40 objective, using the cross sections obtained from different levels of the testis, the seminiferous tubules were selected, and the number of spermatogonia and pachytene spermatocytes was counted on the same seminiferous tubule. With an ×20 objective and Carl Zeiss software (Axio Vision 4.7.2), the microphotography was made. The thickness of the seminiferous epithelium (in µm) was measured, as the distance from the basal lamina to the head of mature spermatozoa.

### 4.14. The Estimation of AMP-Activated Protein Kinase with Western Blotting Analysis

The testis tissues were homogenized in the ratio 1:20 in the lysis buffer containing 20 mM Tris-HCl (pH 7.5), 150 mM sodium chloride, 2 mM EDTA, 2 mM EGTA, 0.5% Triton X-100, 0.25% sodium deoxycholate and 0.02% sodium azide, supplemented with the protease inhibitor cocktail (“Sigma-Aldrich”, St. Louis, MO, USA) and phosphatase inhibitor cocktail PhosStop (“Roche”, Basel, Switzerland). The testes lysates were kept on ice for 1 h until the lysis of the samples was complete. The large fragments of cells and the undamaged cells were separated by centrifugation (10,000× *g*, 10 min, 4 °C). The concentration of protein was measured by the Lowry method with BSA as a standard. Thirty micrograms of protein per sample were run on 10% SDS-polyacrylamide gel, followed by transfer to a nitrocellulose membrane (0.45 μm) (“GE Healthcare, Amersham Biosciences AB”, Buckinghamshire, UK) by electroblotting (100 V, 1 h) in the mini trans-blot module (“Bio-Rad Laboratories Inc.”, Des Plaines, IL, USA). The non-specific binding was blocked in the TBST buffer containing 50 mM Tris-HCl (pH 7.5), 150 mM sodium chloride and 0.1% Tween 20 with the addition of 5% fat-free milk for 30 min at the room temperature. The membranes were incubated at 4 °C overnight with the primary antibodies raised against phospho-AMPK-α(Thr^172^) (1:1000) (#2535, “Cell Signaling Technology” Danvers, MA, USA) and AMPK-α (1:1000) (#2793, “Cell Signaling Technology”, Danvers, MA, USA). The immunostaining was made using the horseradish peroxidase-conjugated anti-mouse (#7076, “Cell Signaling Technology”, Danvers, MA, USA) or anti-rabbit (#7074, “Cell Signaling Technology”, Danvers, MA, USA) immunoglobulins at 1:1000–1:3000 dilution (1 h at the room temperature), and the Novex ECL Chemiluminescent Substrate Reagent Kit (“Invitrogen, Life Technologies”, Waltham, MA, USA). The images were obtained with exposure to the premium X-ray film (“Phenix Research Product”, Candler, NC, USA). To normalize the data, the membranes were treated with the antibodies raised against glyceraldehyde 3-phosphate dehydrogenase (GAPDH) (1:5000) (#NB600-502, “Novus Biologicals”, Centennial, CO, USA). The relative amount of phospho-AMPK-α(Thr^172^) was determined by adjusting for total protein AMPK-α or for GAPDH. The open-source application software Bio7 was used for quantification of the positive bands of the scanned films.

### 4.15. Preparation of the Rat Blood and Tissues Samples and Determination of TP3 Content in Them Using Reverse-Phase HPLC

The blood samples were taken from the rat tail vein under local anesthesia (2% Lidocaine solution, 2–4 mg/kg b.w.) into tubes containing EDTA. To obtain the blood plasma, the samples were centrifuged for 15 min at 750 g (+4 °C). Acetonitrile was added to 0.5 mL of the blood plasma to a ratio of 1:2, after which the mixture was vigorously stirred [133]. Then the mixture was centrifuged for 15 min at 12,000× *g* (+4 °C). For HPLC analysis, the upper phase was taken. The tissue samples from the brain, liver, thyroid gland and testes were obtained after anesthesia of rats with chloral hydrate (400 mg/kg b.w., i.p.). Then the samples from the brain, liver and thyroid gland were weighted and homogenized on ice in four volumes of 50 mM Tris-HCl buffer (pH 7.2). In the case of the testes, 2 mL of the same Tris-HCl buffer was added to the testis samples. Acetonitrile was added to the homogenates of the liver and thyroid gland to a ratio of 1:2, after which the mixture was vigorously stirred. In the case of the brain and testes, 300 μL of the homogenate were taken and mixed with acetonitrile in a ratio 1:4 [133]. Then all homogenate samples were centrifuged for 15 min at 12,000× *g* (+4 °C). The upper phase (supernatant) was taken for HPLC analysis.

Reversed phase HPLC was used to analyze TP3 content in the blood and tissue samples. A liquid chromatograph LC-20 Prominence with a diode array detector SPD-M20A (“Shimadzu”, Kyoto, Japan) and the column Luna C18 (150 × 2.1 mm; 5 um) (“Phenomenex”, Torrance, CA, USA) were used. For separation, a gradient mode (eluent A: 0.05% trifluoroacetic acid in water/eluent B: 0.05% trifluoroacetic acid in acetonitrile; elution mode: 0–2 min, 95% eluent A, 5% eluent B, isocratic; 2–14 min, 95->1% eluent A, 5->99% eluent B, gradient; 14–16 min, 1% eluent A, 99% eluent B, isocratic; 16–17 min, 1->95% eluent A, 99->5% eluent B, gradient; 17–23 min, 95% eluent A, 5% eluent B, isocratic) and the following chromatographic conditions (UV detection wavelength 254 nm; flow rate 0.3 mL/min; column temperature 30 °C; injected sample volume 5 μL) were us.

### 4.16. Statistical Analysis of the Experimental Data

The data were analyzed using the software IBM SPSS Statistics 22 (“IBM”, New York, NY, USA). The multiple comparisons between the groups of rats were assessed statistically using the one-way analysis of variance (ANOVA) and multivariate general linear model analysis with the Tukey post hoc test, and results are presented as the mean ± standard error of the mean (M ± SEM) for metabolic and hormonal parameters, gene expression, sperm analysis, immunohistochemical and morphometric data. All differences are considered as significant at the *p <* 0.05.

## 5. Conclusions

We have shown that a five-week administration of MF at a dose of 120 mg/kg/day (orally) improves testicular steroidogenesis, restores spermatogenesis, and normalizes seminiferous tubule morphology in male rats with HFD/STZ-induced T2DM. In diabetic male rats, MF treatment significantly enhances the steroidogenic effects of single-dose LH/hCG-R-agonists, such as hCG and 5-amino-*N-tert*-butyl-2-(methylsulfanyl)-4-(3-(nicotinamido)phenyl))thieno[2,3-*d*]-pyrimidine-6-carboxamide (TP3), differing in their mechanisms of action on LH/hCG-R. This is due to a number of causes, including MF-mediated maintenance of LH/hCG-R expression in the testicular Leydig cells, restoration of seminiferous tubule ultrastructure, and inhibition of aromatase expression and estradiol production in the testes. The improving effect of MF therapy on the steroidogenic activity of hCG and TP3 can be used in clinical practice to enhance the response of the testes and ovaries to single-dose treatment with the orthosteric (hCG, LH) and allosteric (low-molecular-weight heterocyclic compounds, including thieno[2,3-*d*]-pyrimidines) LH/hCG-R-agonists.

At the same time, after long-term (for five days) administration of the investigated LH/hCG-R-agonists to MF-treated diabetic rats, their steroidogenic and spermatogenic effects diminished, and this occurred in the conditions of considerable restoration of androgens production and significant improvement of spermatogenesis induced by long-term MF therapy. One of the reasons for this is a significant decrease in the gene expression of LH/hCG-R and its content in the testes of MF-treated diabetic rats, which were injected with LH/hCG-R-agonists for five days. In this case, LH/hCG-R expression was reduced in comparison with a single administration of LH/hCG-R-agonists to MF-treated rats and with diabetic rats that received LH/hCG-R-agonist injections for five days. Along with this, in MF-treated rats, a five-day administration of LH/hCG-R-agonists abolished the inhibitory effect of these agonists, observed after their single administration, on aromatase gene expression and, thereby, contributed to a decrease in the testosterone/estradiol ratio, making an additional contribution to the weakening of androgen-stimulating effect of long-term administration of hCG and TP3.

We assume that since steroidogenic and spermatogenic functions of the testes are largely restored in MF-treated rats, prolonged administration of LH/hCG-R-agonists can lead to hyperactivation of testicular steroidogenesis. To avoid this, the compensatory mechanisms are triggered in the testes (a pronounced decrease in LH/hCG-R expression, an increase in the conversion of androstenedione to estradiol), which are designed to prevent such hyperactivation. A weakening of the steroidogenic effects of LH/hCG-R-agonists may be due to the need to protect spermatozoa from an excess of androgens, which negatively affects their fertility. There is evidence of increased apoptosis in testicular cells after long-term administration of high doses of hCG to rodents [134,135]. Although we use a relatively low dose of gonadotropin, in MF-treated rats, we cannot exclude the appearance of a pro-apoptotic effect of hCG on testicular cells. The effect of low-molecular-weight allosteric LH/hCG-R-agonists on apoptosis and autophagy in the testes, including in diabetes and MF therapy, has not been studied at all. The study of these issues should be the subject of our further research. The data obtained suggest that under the conditions of MF therapy, which considerably restores male reproduction in T2DM, long-term use of gonadotropins and allosteric LH/hCG-R-agonists may be ineffective and even cause negative consequences. However, additional studies are needed to confirm this hypothesis.

## Figures and Tables

**Table 1:** The body weight, blood levels of glucose, insulin, leptin, glycated hemoglobin (HbA1c) and lipids, and the insulin resistance index in the control, diabetic and metformin-treated diabetic male rats.

Parameter	Control, *n* = 30	Diabetes, *n* = 30	Diabetes + MF, *n* = 30
Body weight, g	352.2 ± 3.9	402.2 ± 5.2 ^a^	361.3 ± 5.5 ^b^
Fasting glucose, mM	4.34 ± 0.08	6.57 ± 0.12 ^a^	5.64 ± 0.11 ^a,b^
Glucose (120′, GTT), mM	5.37 ± 0.09	9.77 ± 0.26 ^a^	6.48 ± 0.19 ^a,b^
HbA1c, %	4.52 ± 0.08	7.07 ± 0.14 ^a^	5.56 ± 0.14 ^a,b^
Fasting insulin, ng/mL	0.61 ± 0.04	0.96 ± 0.05 ^a^	0.66 ± 0.04 ^b^
Insulin (120, GTT), ng/mL	0.77 ± 0.07	1.30 ± 0.09 ^a^	0.86 ± 0.06 ^b^
IR (fasting), rel.units	2.68 ± 0.18	6.41 ± 0.42 ^a^	3.80 ± 0.28 ^a,b^
IR (120, GTT), rel.units	4.21 ± 0.40	13.19 ± 1.22 ^a^	5.69 ± 0.48 ^b^
Fasting leptin, ng/mL	1.81 ± 0.09	3.49 ± 0.11 ^a^	2.00 ± 0.07 ^b^
Leptin (120, GTT), ng/mL	2.51 ± 0.13	4.70 ± 0.18 ^a^	3.17 ± 0.15 ^a,b^
Triglycerides, mM	0.75 ± 0.01	1.18 ± 0.05 ^a^	0.84 ± 0.03 ^b^
Total cholesterol, mM	4.31 ± 0.06	6.38 ± 0.11 ^a^	4.91 ± 0.08 ^a,b^
Testosterone, nM	12.01 ± 0.70	7.02 ± 0.39 ^a^	8.82 ± 0.43 ^a,b^

Note. The duration of MF treatment (120 mg/kg/day) was four weeks (before treatment of animals with LH/hCG-R-agonists). ^a^—the differences with control rats are significant at *p* < 0.05; ^b^—the differences between the untreated and MF-treated diabetic rats are significant at *p* < 0.05. The data are presented as the M ± SEM, *n* = 30.

**Table 2:** The effects of five-week metformin treatment and a single dose of TP3 and hCG on the blood testosterone levels in the control and diabetic animals.

Group	Testosterone Concentration, nM			
	0 (before)	1 (11 a.m.)	3 (1 p.m.)	5 (3 p.m.)
C1	13.53 ± 1.45	16.34 ± 2.52	17.66 ± 2.82	14.96 ± 1.60
CT1	11.11 ± 1.42	23.52 ± 3.43	34.58 ± 3.82 ^c^	33.35 ± 3.89 ^c^
CG1	13.14 ± 1.42	35.36 ± 3.17 ^d,e^	70.88 ± 4.77 ^d,e^	62.20 ± 1.71 ^d,e^
D1	5.69 ± 0.80 ^a^	7.20 ± 0.68	6.72 ± 0.47 ^a^	7.08 ± 0.84 ^a^
DT1	5.73 ± 0.64 ^f^	10.06 ± 0.73 ^f^	19.02 ± 1.98 ^c,f^	22.28 ± 1.75 ^c,f^
DG1	6.98 ± 0.58 ^g^	19.59 ± 2.11 ^d,e,g^	39.01 ± 2.08 ^d,e,g^	32.70 ± 2.53 ^d,e,g^
DM1	8.99 ± 0.89 ^a^	16.40 ± 3.64	11.85 ± 0.96	10.44 ± 1.39
DMT1	9.61 ± 1.36	21.94 ± 2.21 ^h^	29.68 ± 2.29 ^c,h^	33.03 ± 1.40 ^c,h^
DMG1	9.59 ± 0.76	32.70 ± 2.41 ^d,e,h^	60.74 ± 3.90 ^d,e,h^	58.13 ± 1.12 ^d,e,h^

Note. The duration of MF treatment (120 mg/kg/day) was five weeks, and the single doses of TP3 and hCG were 15 mg/kg and 15 IU/rat, respectively. A single injection of LH/hCG-R-agonists was carried out on the 35th day of MF treatment (the final day of the experiment). ^a^—the difference between the C1 vs. D1 or DM1; ^c^—the difference between the C1 vs. CT1, D1 vs. DT1 and DM1 vs. DMT1; ^d^—the difference between the C1 vs. CG1, D1 vs. DG1 and DM1 vs. DMG1; ^e^—the difference between the CT1 vs. CG1, DT1 vs. DG1 and DMT1 vs. DMG1; ^f^—the difference between the CT1 vs. DT1; ^g^—the difference between the CG1 vs. DG1; and ^h^—the difference between the DT1 vs. DMT1 and DG1 vs. DMG1 are significant at *p* < 0.05. The data are presented as the M ± SEM, *n* = 5.

**Table 3:** The effects of five-week metformin treatment and five-day administration of TP3 and hCG on the blood testosterone levels in the control and diabetic animals.

Group	Testosterone Concentration, nM					
	0 (before)	1st Day	2nd Day	3rd Day	4th Day	5th Day
C5	12.52 ± 1.55	12.45 ± 1.07	15.18 ± 1.07	13.40 ± 1.68	11.55 ± 1.35	12.64 ± 1.57
CT5	12.30 ± 1.56	32.72 ± 4.26 ^c^	35.30 ± 5.61 ^c^	31.87 ± 4.19 ^c^	32.20 ± 2.95 ^c^	33.96 ± 3.94 ^c^
CG5	13.03 ± 1.73	72.66 ± 5.89 ^d,e^	33.11 ± 2.32 ^d^	34.14 ± 3.72 ^d^	40.09 ± 1.09 ^d,e^	39.48 ± 2.95 ^d^
D5	6.62 ± 0.79 ^a^	6.67 ± 1.34 ^a^	6.88 ± 0.89 ^a^	6.06 ± 0.99 ^a^	6.61 ± 0.43 ^a^	6.37 ± 0.94 ^a^
DT5	6.89 ± 0.92 ^f^	19.32 ± 1.49 ^c,f^	27.40 ± 4.48 ^c^	26.32 ± 4.76 ^c^	27.41 ± 4.67 ^c^	29.93 ± 3.80 ^c,f^
DG5	6.27 ± 1.03 ^g^	44.84 ± 4.67 ^d,e,g^	33.15 ± 5.89 ^d^	29.19 ± 3.47 ^d^	32.16 ± 6.18 ^d^	35.37 ± 5.86 ^d^
DM5	9.91 ± 0.40	8.71 ± 0.83 ^a^	10.81 ± 0.92 ^a,b^	12.59 ± 1.72 ^b^	10.65 ± 0.81 ^b^	11.11 ± 1.23
DMT5	10.38 ± 1.27	30.41 ± 2.52 ^c^	29.77 ± 2.18 ^c^	25.42 ± 2.80 ^c^	26.42 ± 2.38 ^c^	23.62 ± 3.15 ^c^
DMG5	12.06 ± 2.13	65.37 ± 7.09 ^d,e^	38.06 ± 5.70 ^d^	28.77 ± 2.66 ^d^	28.55 ± 2.40 ^d^	27.92 ± 2.37 ^d^

Note. The duration of MF treatment (120 mg/kg/day) was five weeks, and the daily doses of TP3 and hCG were 15 mg/kg and 15 IU/rat, respectively. Five-day treatment with TP3 and hCG was carried out from the 31st to the 35th days of MF treatment. ^a^—the difference between the C5 vs. D5 or DM5, ^b^—the difference between the D5 vs. DM5; ^c^—the difference between the C5 vs. CT5, D5 vs. DT5 and DM5 vs. DMT5; ^d^—the difference between the C5 vs. CG5, D5 vs. DG5 and DM5 vs. DMG5; ^e^—the difference between the CT5 vs. CG5, DT5 vs. DG5 and DMT5 vs. DMG5; ^f^—the difference between the CT5 vs. DT5; and ^g^—the difference between the CG5 vs. DG5 are significant at *p* < 0.05. The data are presented as the M ± SEM, *n* = 5.

**Table 4:** The influence of a single dose of hCG and TP3 on the testicular levels of testosterone, estradiol and their precursors in the control and diabetic rats, and the effect of five-week metformin treatment.

Group	Progestrerone, nmol/g	17-OH-Progesterone, pmol/g	Androstenedione, ng/g	Testosterone, nmol/g	Estradiol, pmol/g
C1	0.314 ± 0.018	61.92 ± 10.00	59.07 ± 7.50	1.079 ± 0.045	49.39 ± 2.97
CT1	0.480 ± 0.036	70.81 ± 3.64	76.20 ± 13.27	1.522 ± 0.103	44.00 ± 2.26
CG1	1.312 ± 0.085 ^d,e^	119.98 ± 13.37 ^d,e^	90.48 ± 7.35	1.830 ± 0.214 ^d^	35.82 ± 1.69 ^d^
D1	0.283 ± 0.017	50.30 ± 5.33	59.32 ± 4.46	0.716 ± 0.061 ^a^	57.18 ± 2.53
DT1	0.419 ± 0.049	77.70 ± 13.02	60.28 ± 9.49	1.297 ± 0.177 ^c^	36.56 ± 5.21 ^c^
DG1	0.717 ± 0.070 ^d,e,g^	79.69 ± 8.10 ^g^	62.22 ± 6.11	1.456 ± 0.138 ^d^	35.93 ± 2.50 ^d^
DM1	0.285 ± 0.019	54.23 ± 10.92	69.93 ± 10.25	0.999 ± 0.149	39.08 ± 2.48 ^a,b^
DMT1	0.355 ± 0.032	72.03 ± 5.96	92.03 ± 8.74	1.681 ± 0.152	35.09 ± 3.71
DMG1	0.808 ± 0.092 ^d,e,g^	123.05 ± 8.65 ^d,e,h^	114.27 ± 15.48 ^h^	2.373 ± 0.341 ^d^	29.24 ± 2.38

Note. The duration of MF treatment (120 mg/kg/day) was five weeks. The samples to measure the content of testosterone, estradiol and their precursors in the testes were taken 5 h after the injection of LH/hCG-R-agonists. ^a^^—^the difference between the C1 vs. D1 or DM1; ^b^—the difference between the D1 vs. DM1; ^c^—the difference between the D1 vs. DT1; ^d^—the difference between the C1 vs. CG1, D1 vs. DG1 and DM1 vs. DMG1; ^e^—the difference between the CT1 vs. CG1, DT1 vs. DG1 and DMT1 vs. DMG1; ^g^—the difference between the CG1 vs. DG1 or DMG1; and ^h^—the difference between the DG1 vs. DMG1 are significant at *p* < 0.05. The data are presented as the M ± SEM, *n* = 5.

**Table 5:** The influence of five-day treatment with hCG and TP3 on the testicular levels of testosterone, estradiol and their precursors in the control and diabetic rats, and the effect of five-week metformin treatment.

Group	Progestrerone, nmol/g	17-OH-Progesterone, pmol/g	Androstenedione, ng/g	Testosterone, nmol/g	Estradiol, pmol/g
C5	0.325 ± 0.023	69.0 ± 6.9	60.8 ± 5.5	1.150 ± 0.095	51.25 ± 3.50
CT5	0.463 ± 0.090	111.5 ± 10.8 ^c^	120.1 ± 13.9 ^c^	1.597 ± 0.153	50.57 ± 1.32
CG5	0.743 ± 0.064 ^d,e^	211.6 ± 13.0 ^d,e^	158.7 ± 16.8 ^d^	1.723 ± 0.103 ^d^	37.81 ± 6.25
D5	0.262 ± 0.025	51.7 ± 4.9	61.3 ± 4.8	0.676 ± 0.080 ^a^	55.95 ± 1.51
DT5	0.333 ± 0.036	78.1 ± 9.2	120.0 ± 15.2 ^c^	1.462 ± 0.077 ^c^	48.92 ± 4.17
DG5	0.688 ± 0.125 ^d,e^	355.7 ± 38.2 ^d,e,g^	190.5 ± 9.6 ^d,e^	1.598 ± 0.111 ^d^	39.13 ± 3.57 ^d^
DM5	0.287 ± 0.021	60.8 ± 4.7	63.5 ± 6.0	1.265 ± 0.155 ^b^	36.37 ± 5.28 ^a,b^
DMT5	0.322 ± 0.032	77.1 ± 6.7 ^f^	122.4 ± 23.6 ^c^	1.170 ± 0.107	55.28 ± 3.72 ^c^
DMG5	0.858 ± 0.128 ^d,e^	370.1 ± 31.2 ^d,e,g^	167.5 ± 6.0 ^d^	1.469 ± 0.151	45.98 ± 3.79

Note. The duration of MF treatment (120 mg/kg/day) was five weeks. The samples to measure the content of testosterone, estradiol and their precursors in the testes were taken 5 h after the last injection of LH/hCG-R-agonist. ^a^—the difference between the C5 vs. D5 or DM5, ^b^—the difference between the D5 vs. DM5; ^c^—the difference between the C5 vs. CT5, D5 vs. DT5 and DM5 vs. DMT5; ^d^—the difference between the C5 vs. CG5, D5 vs. DG5 and DM5 vs. DMG5; ^e^—the difference between the CT5 vs. CG5, DT5 vs. DG5 and DMT5 vs. DMG5; ^f^—the difference between the CT5 vs. DMT5; and ^g^—the difference between the CG5 vs. DG5 or DMG5 are significant at *p* < 0.05. The data are presented as the M ± SEM, *n* = 5.

**Table 6:** The effects of five-week metformin treatment, five-day treatment with LH/hCG-R-agonists and their combination on thickness of the seminiferous epithelium and the number of spermatogonia and pachytene spermatocytes in the control and diabetic rats.

Group	Thickness of the Seminiferous Epithelium, μm	Number of Spermatogonia, Units	Number of Pachytene Spermatocytes, Units
C5	66.20 ± 0.92	54.06 ± 1.19	49.74 ± 0.84
CT5	68.35 ± 0.84	57.64 ± 0.92 ^c^	53.96 ± 0.75 ^c^
CG5	67.65 ± 0.99	62.02 ± 0.89 ^d,e^	56.34 ± 0.74^d^
D5	58.38 ± 0.71 ^a^	47.04 ± 0.86 ^a^	37.28 ± 0.77 ^a^
DT5	69.97 ± 1.04 ^c^	57.72 ± 1.05 ^c^	51.26 ± 0.89 ^cf^
DG5	66.65 ± 1.10 ^d,e^	60.82 ± 0.89 ^d^	53.80 ± 1.24 ^d^
DM5	62.68 ± 1.14 ^a,b^	59.08 ± 0.77 ^a,b^	49.54 ± 1.05 ^b^
DMT5	66.44 ± 1.07 ^c,h^	59.36 ± 0.64	50.12 ± 0.74 ^f^
DMG5	64.79 ± 0.70	58.76 ± 0.83 ^g^	51.28 ± 0.79 ^g^

Note. The number of spermatogonia and the number of pachytene spermatocytes are represented as the number of cells per one seminiferous tubule. ^a^—the difference between the C5 vs. D5 or DM5; ^b^—the difference between the D5 vs. DM5; ^c^—the difference between the C5 vs. CT5, D5 vs. DT5 and DM5 vs. DMT5; ^d^—the difference between the C5 vs. CG5 and D5 vs. DG5; ^e^—the difference between the CT5 vs. CG5 and DT5 vs. DG5; ^f^—the difference between the CT5 vs. DT5 or DMT5; ^g^—the difference between the CG5 vs. DMG5; and ^h^—the difference between the DT5 vs. DMT5 are significant at *p* < 0.05. The data are presented as the M ± SEM, *n* = 5.

**Table 7:** The ratios of Thr^172^-phosphorylated α-AMPK/total α-AMPK, Thr^172^-phosphorylated α-AMPK/GAPDH and total α-AMPK/GAPDH in the testes of control and diabetic rats, and the effects of the treatment with MF and LH/hCG-R-agonists.

Group	pAMPKα(Thr^172^)/ tAMPKα	pAMPKα(Thr^172^)/ GAPDH	tAMPKα/GAPDH
C5	0.502 ± 0.045	0.884 ± 0.091	1.862 ± 0.324
CT5	0.491 ± 0.071	0.781 ± 0.036	1.717 ± 0.233
CG5	0.541 ± 0.113	0.825 ± 0.107	1.707 ± 0.292
D5	0.687 ± 0.085	0.731 ± 0.065	1.093 ± 0.102
DT5	0.773 ± 0.111	0.780 ± 0.067	1.076 ± 0.161
DG5	0.864 ± 0.092	0.807 ± 0.100	1.000 ± 0.185
DM5	1.081 ± 0.203 ^a^	0.868 ± 0.051	0.917 ± 0.180 ^a^
DMT5	0.771 ± 0.113	0.909 ± 0.147	1.306 ± 0.320
DMG5	0.502 ± 0.067 ^d,h^	0.680 ± 0.073	1.443 ± 0.189

Note. ^a^—the difference between the C5 vs. D5 or DM5; ^d^—the difference between the DM5 vs. DMG5; and ^h^—the difference between the DG5 vs. DMG5 are significant at *p* < 0.05. The data are presented as the M ± SEM, *n* = 5.

**Table 8:** The primers for the study of target and reference genes using real-time PCR analysis.

Genes	Forward/Reverse Sequence	PS	AT	Genbank
*Lhr*	(For) CTGCGCTGTCCTGGCC	103	55	NM_012978.1
	(Rev) CGACCTCATTAAGTCCCCTGAA			
*Star*	(For) AAGGCTGGAAGAAGGAAAGC	66	55	NM_031558.3
	(Rev) CACCTGGCACCACCTTACTT			
*Cyp11a1*	(For) TATTCCGCTTTGCCTTTGAG	74	55	NM_017286.3
	(Rev) CACGATCTCCTCCAACATCC			
*Hsd3b*	(For) AGGCCTGTGTCCAAGCTAGTGT	161	55	XM_017591325.1
	(Rev) CTCGGCCATCTTTTTGCTGTAT			
*Cyp17a1*	(For) CATCCCCCACAAGGCTAAC	61	55	XM_006231435.3
	(Rev) TGTGTCCTTGGGGACAGTAAA			
*Hsd17b*	(For) CCTTTGGCTTTGCCATGAGA	68	55	NM_024392.2
	(Rev) CAATCCATCCTGCTCCAACCT			
*Cyp19a1*	(For) GGTATCAGCCTGTCGTGGAC	118	56	NM_017085.2
	(Rev) AGCCTGTGCATTCTTCCGAT			
*Actb*	(For) CTGGCACCACACCTTCTACA	125	55	NM_031144.3
	(Rev) AGGTCTCAAACATGATCTGGGT			

Note. PS—product size (bp), AT—annealing temperature (°C).

## Data Availability

Data is contained within the article or Supplementary Materials.

## Supplementary Materials

The following are available online at https://www.mdpi.com/article/10.3390/ijms23010198/s1.

## Abbreviations

CYP11A1 (gene *Cyp**11a1*)	cytochrome P450_scc_
CYP17A1 (gene *Cyp**17a1*)	cytochrome P450 17A1/steroid 17α-monooxygenase
CYP19A1 (gene *Cyp19a1*)	cytochrome P450A1 (Aromatase)
hCG	human chorionic gonadotropin
HFD	high-fat diet
3β-HSD (gene *Hsd**3b*)	3β-hydroxysteroid dehydrogenase
17β-HSD (gene *Hsd17b*)	17β-hydroxysteroid dehydrogenase
LH	luteinizing hormone
LH/hCG-R	luteinizing hormone/human chorionic gonadotropin receptor
MF	Metformin
StAR	steroidogenic acute regulatory protein
STZ	Streptozotocin
T2DM	type 2 diabetes mellitus
TP3	5-amino-*N-tert*-butyl-2-(methylsulfanyl)-4-(3-(nicotinamido)phenyl)thieno[2,3-*d*] pyrimidine-6-carboxamide
