# Supplementary material for: The Effects of Separate and Combined Treatment of Male Rats with Type 2 Diabetes with Metformin and Orthosteric and Allosteric Agonists of Luteinizing Hormone Receptor on Steroidogenesis and Spermatogenesis
Source: Int J Mol Sci. 2021 Dec 24;23(1):198. doi: 10.3390/ijms23010198 (PMC8745465; doi:10.3390/ijms23010198)
Supplement: Supplementary file 1 [file ijms-23-00198-s001.zip › Table S6.pdf]

Table S6. The ratios of steroid hormones in the testes of control, diabetic and MF-treated diabetic rats, and the effect of five-day administration of TP3 and hCG.

Group	Progesterone/ testosterone	17-0H-Progesterone/ testosterone	Androstenedione/ testosterone	Testosterone/ estradiol
C5	0.026 ± 0.002	5.53 ± 0.25	4.89 ± 0.19	0.245 ± 0.018
CT5	0.014 ± 0.002 [c]	3.35 ± 0.31 [c]	3.56 ± 0.27 [c]	0.673 ± 0.079
CG5	0.019 ± 0.001 [d]	5.39 ± 0.21 [e]	3.99 ± 0.17 [d]	1.180 ± 0.229 [d]
D5	0.043 ± 0.003 [a]	8.47 ± 0.67 [a]	10.12 ± 0.88 [a]	0.115 ± 0.018 [a]
DT5	0.011 ± 0.001 [c]	2.68 ± 0.32 [c]	4.12 ± 0.52 [c]	0.642 ± 0.116 [c]
DG5	0.019 ± 0.001 [de]	10.55 ± 0.81 [eg]	5.89 ± 0.79 [d]	0.931 ± 0.164 [d]
DM5	0.026 ± 0.001 [b]	5.57 ± 0.34 [b]	5.76 ± 0.27 [b]	0.317 ± 0.027 [b]
DMT5	0.014 ± 0.002 [c]	3.49 ± 0.49 [c]	5.18 ± 0.60	0.430 ± 0.059
DMG5	0.030 ± 0.002 [egh]	13.29 ± 0.41 [degh]	6.12 ± 0.40 [g]	0.616 ± 0.048 [de]

[a] – the difference between the C5 *vs*. D5; [b] – the difference between the D5 *vs*. DM5; [c] – the difference between the C5 *vs*. CT5, D5 *vs*. DT5 and DM5 *vs*. DMT5; [d] – the difference between the C5 *vs*. CG5, D5 *vs*. DG5 and DM5 *vs*. DMG5; [e] – the difference between the CT5 *vs*. CG5, DT5 *vs*. DG5 and DMT5 *vs*. DMG5; [g] – the difference between the CG5 *vs*. DG5 or DMG5; and [h] – the difference between the DG5 *vs*. DMG5 are significant at $p<0.05$. The data are presented as the $M \pm SEM$, n=5.
