# Supplementary material for: The Effects of Separate and Combined Treatment of Male Rats with Type 2 Diabetes with Metformin and Orthosteric and Allosteric Agonists of Luteinizing Hormone Receptor on Steroidogenesis and Spermatogenesis
Source: Int J Mol Sci. 2021 Dec 24;23(1):198. doi: 10.3390/ijms23010198 (PMC8745465; doi:10.3390/ijms23010198)
Supplement: Supplementary file 1 [file ijms-23-00198-s001.zip › Figure S1.pdf]

A

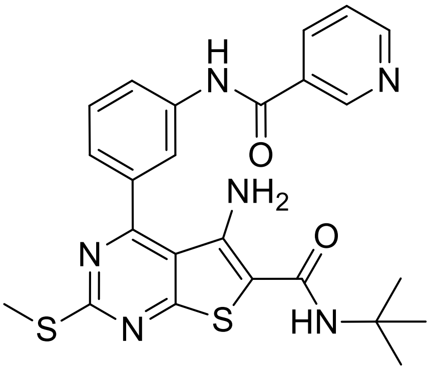

B

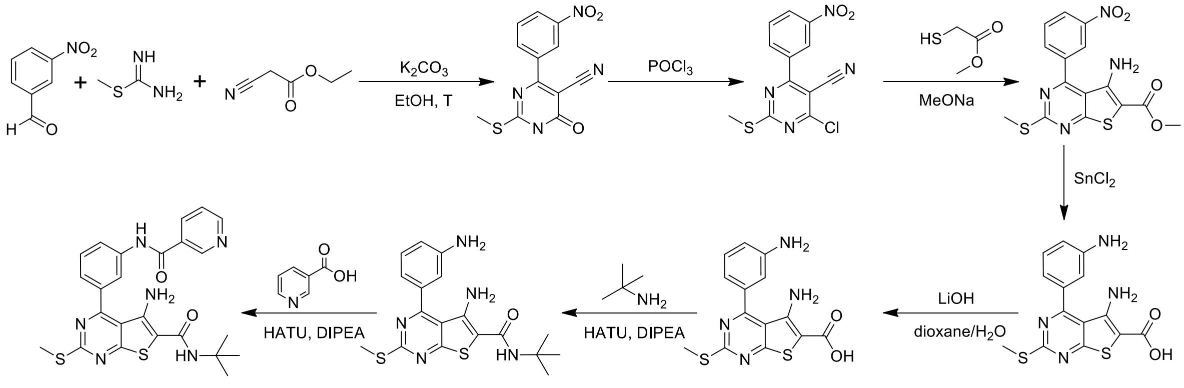

K2CO3
EtOH, T
POCl3
MeONa
SnCl2
LiOH
dioxane/H2O
HATU, DIPEA
HATU, DIPEA

TP- 3

C

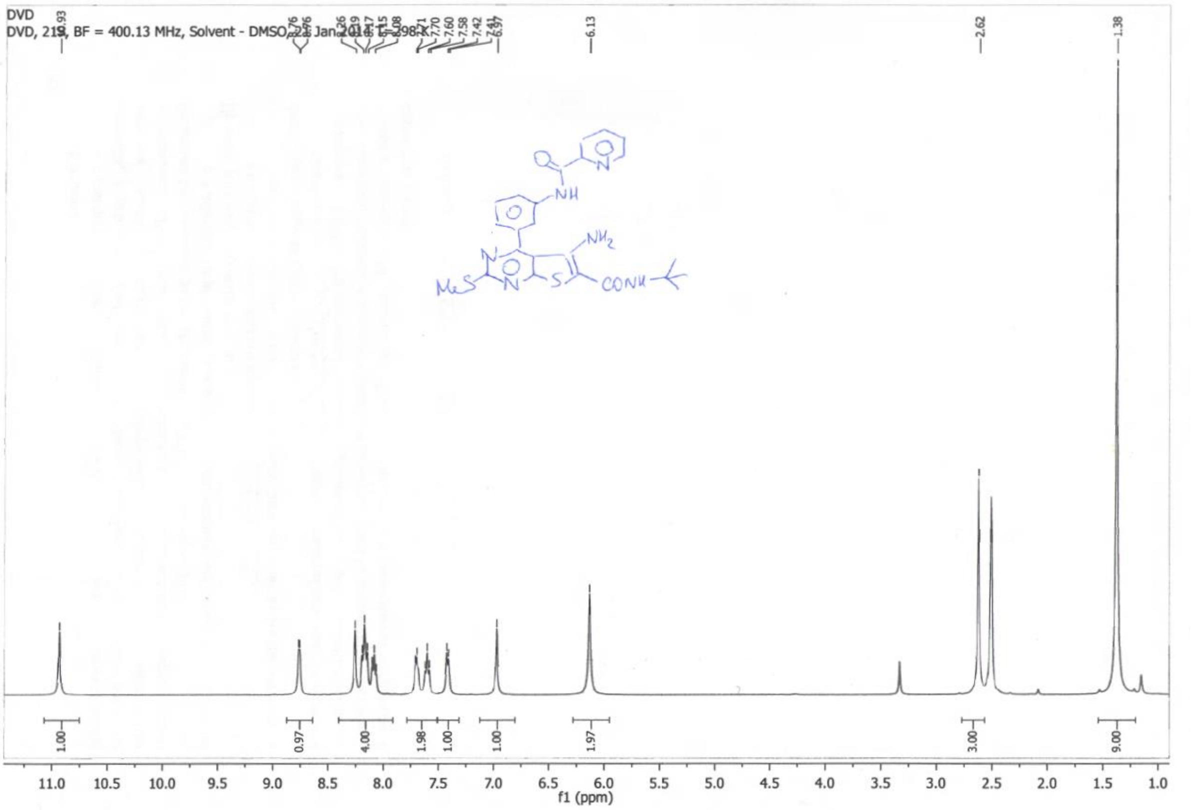

DVD
DVD, 219, BF = 400.13 MHz, Solvent - DMSO, 23 Jan 2016, T=298 K
f1 (ppm)

D

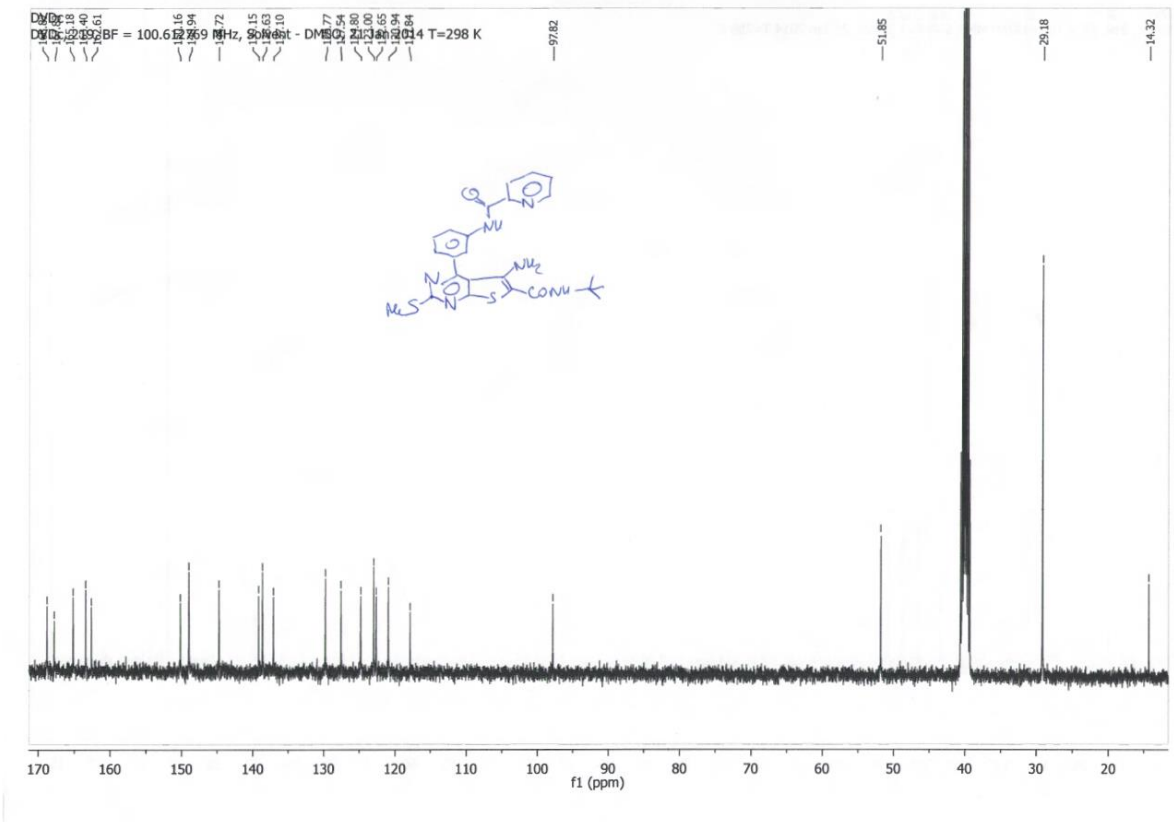

E

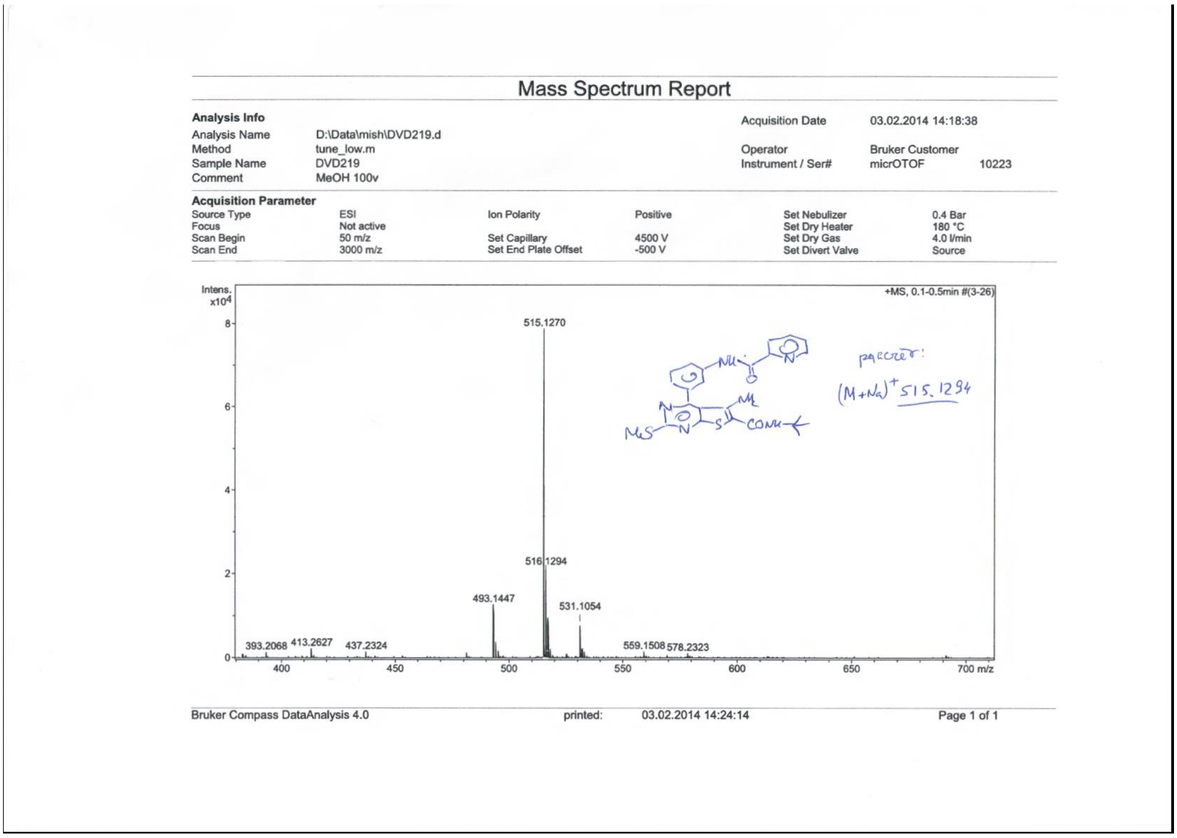

Figure S1. The structure of 5-amino-*N*-*tert*-butyl-2-(methylsulfanyl)-4-(3-(nicotinamido)phenyl)thieno[2,3-*d*] pyrimidine-6-carboxamide (TP3) (**A**), its synthesis (**B**), the ^{1}H-NMR (**C**) and ^{13}C-NMR spectra (**D**) and the mass spectra (**E**) of this compound.
