# Supplementary material for: The Effects of Separate and Combined Treatment of Male Rats with Type 2 Diabetes with Metformin and Orthosteric and Allosteric Agonists of Luteinizing Hormone Receptor on Steroidogenesis and Spermatogenesis
Source: Int J Mol Sci. 2021 Dec 24;23(1):198. doi: 10.3390/ijms23010198 (PMC8745465; doi:10.3390/ijms23010198)
Supplement: Supplementary file 1 [file ijms-23-00198-s001.zip › Table S1.pdf]

Table S1. The effect of five-day treatment with TP3 and hCG on the body, fat and testes weight, the gonadosomatic index (GSI) and lipids concentration in the control rats and the untreated and MF-treated diabetic animals.

Group	Body weight, g	Fat weight, g	Testes weight, g	GSI, arb. units	Triglycerides, mM	Cholesterol, mM
C5	369.6±7.4	8.5±0.5	3.82±0.07	1.04±0.03	0.73±0.04	4.23±0.19
CT5	362.0±9.0	8.2±0.4	3.96±0.10	1.09±0.01	0.75±0.05	4.18±0.09
CG5	362.6±10.7	8.1±0.3	4.10±0.11	1.13±0.03	0.69±0.03	4.14±0.16
D5	420.4±14.2 [a]	15.5±2.0 [a]	3.72±0.06	0.89±0.04	1.27±0.08 [a]	6.46±0.22 [a]
DT5	414.4±17.9 [a]	15.2±2.3 [a]	3.88±0.09	0.94±0.05	1.25±0.12 [a]	6.35±0.29 [a]
DG5	412.2±7.9 [a]	14.7±1.1 [a]	3.94±0.10	0.96±0.04	1.10±0.07 [a]	6.23±0.14 [a]
DM5	369.0±12.3 [b]	10.1±0.8 [b]	3.74±0.05	1.02±0.02	0.79±0.03 [b]	4.67±0.22 [b]
DMT5	372.2±15.4	10.7±1.3	3.82±0.07	1.04±0.06	0.86±0.09 [b]	4.98±0.27 [b]
DMG5	368.2±14.0 [b]	10.6±0.9	3.94±0.08	1.07±0.04	0.96±0.08 [a,b]	4.89±0.21 [b]

Note. The duration of MF treatment (120 mg/kg/day) was 5 weeks. [a] – the difference between the C5 *vs.* D5 or DM5, [b] – the difference between the D5 *vs.* DM5; [c] – the difference between the C5 *vs.* CT5, the D5 *vs.* DT5 and the DM5 *vs.* DMT5; [d] – the difference between the C5 *vs.* CG5, the D5 *vs.* DG5 and the DM5 *vs.* DMG5; [e] – the difference between the CT5 *vs.* CG5, the DT5 *vs.* DG5 and the DMT5 *vs.* DMG5; [f] – the difference between the CT5 *vs.* DT5 or DMT5; [g] – the difference between the CG5 *vs.* DG5 or DMG5; and [h] – the difference between the DT5 *vs.* DMT5 and the DG5 *vs.* DMG5 are significant at $p<0.05$. The data are presented as the $M \pm SEM$, $n=5$.
