# Supplementary material for: The Effects of Separate and Combined Treatment of Male Rats with Type 2 Diabetes with Metformin and Orthosteric and Allosteric Agonists of Luteinizing Hormone Receptor on Steroidogenesis and Spermatogenesis
Source: Int J Mol Sci. 2021 Dec 24;23(1):198. doi: 10.3390/ijms23010198 (PMC8745465; doi:10.3390/ijms23010198)
Supplement: Supplementary file 1 [file ijms-23-00198-s001.zip › Table S2.pdf]

Table S2. The effect of five-day treatment with TP3 and hCG on the blood levels of postprandial glucose, HbA1c, insulin and leptin in the control rats and the untreated and MF-treated diabetic animals.

Group	Glucose, mM	HbA1c, %	Insulin, ng/ml	Leptin, ng/ml
C5	5.18±0.11	4.50±0.12	0.82±0.11	2.19±0.25
CT5	5.20±0.17	4.62±0.18	0.77±0.12	2.58±0.24
CG5	5.26±0.13	4.80±0.16	0.85±0.17	2.40±0.26
D5	8.22±0.51 [a]	6.88±0.35 [a]	1.31±0.13 [a]	5.15±0.26 [a]
DT5	8.64±0.54 [a]	6.96±0.35 [a]	1.46±0.28	5.00±0.37 [a]
DG5	8.32±0.59 [a]	6.86±0.30 [a]	1.33±0.23	4.83±0.33 [a]
DM5	6.14±0.20 [a,b]	5.52±0.17 [a,b]	0.89±0.13	3.13±0.27 [a,b]
DMT5	6.72±0.27 [a,b]	5.46±0.29 [a,b]	0.86±0.09 [b]	3.29±0.55 [b]
DMG5	6.70±0.42 [a]	5.48±0.23 [a,b]	0.97±0.12	3.01±0.28 [b]

Note. The duration of MF treatment (120 mg/kg/day) was 5 weeks. [a] – the difference between the C5 *vs.* D5 or DM5, [b] – the difference between the D5 *vs.* DM5; [c] – the difference between the C5 *vs.* CT5, the D5 *vs.* DT5 and the DM5 *vs.* DMT5; [d] – the difference between the C5 *vs.* CG5, the D5 *vs.* DG5 and the DM5 *vs.* DMG5; [e] – the difference between the CT5 *vs.* CG5, the DT5 *vs.* DG5 and the DMT5 *vs.* DMG5; [f] – the difference between the CT5 *vs.* DT5 or DMT5; [g] – the difference between the CG5 *vs.* DG5 or DMG5; and [h] – the difference between the DT5 *vs.* DMT5 and the DG5 *vs.* DMG5 are significant at $p<0.05$. The data are presented as the $M \pm SEM$, $n=5$.
