# Supplementary material for: The Effects of Separate and Combined Treatment of Male Rats with Type 2 Diabetes with Metformin and Orthosteric and Allosteric Agonists of Luteinizing Hormone Receptor on Steroidogenesis and Spermatogenesis
Source: Int J Mol Sci. 2021 Dec 24;23(1):198. doi: 10.3390/ijms23010198 (PMC8745465; doi:10.3390/ijms23010198)
Supplement: Supplementary file 1 [file ijms-23-00198-s001.zip › Table S3.pdf]

Table S3. The concentration of intraperitoneally injected TP3 in the blood of male rats, assessed for 6 hours (360 min) after injection of TP3 (15 mg/kg, i.p., in DMSO).

Drug	30 min	60 min	120 min	180 min	270 min	360 min
	Drug concentration in the blood, mkg/ml					
TP3	0.351±0.023	0.481±0.025	0.528±0.018	0.476±0.014	0.341±0.012	0.269±0.010

The data are presented as the *M* ± *SEM*, n=5.
