# Supplementary material for: The Effects of Separate and Combined Treatment of Male Rats with Type 2 Diabetes with Metformin and Orthosteric and Allosteric Agonists of Luteinizing Hormone Receptor on Steroidogenesis and Spermatogenesis
Source: Int J Mol Sci. 2021 Dec 24;23(1):198. doi: 10.3390/ijms23010198 (PMC8745465; doi:10.3390/ijms23010198)
Supplement: Supplementary file 1 [file ijms-23-00198-s001.zip › Table S4.pdf]

Table S4. The TP3 content in the testes (A), thyroid (B), liver (C) and brain (D) of male rats, assessed for 6 hours after injection of TP3 (15 mg/kg, i.p., in DMSO).

Drug	Testes	Thyroid	Liver	Brain
	Drug concentration in the tissues, mkg/g of the tissue			
TP3	2.07±0.12	1.92±0.15	2.81±0.23^{a,b}	1.43±0.11^{a,b,c}

The difference with the testes (^{a}), thyroid (^{b}) and liver (^{c}) are significant at $p<0.05$. The data are presented as the $M \pm SEM$, n=5.
