# Supplementary material for: The Effects of Separate and Combined Treatment of Male Rats with Type 2 Diabetes with Metformin and Orthosteric and Allosteric Agonists of Luteinizing Hormone Receptor on Steroidogenesis and Spermatogenesis
Source: Int J Mol Sci. 2021 Dec 24;23(1):198. doi: 10.3390/ijms23010198 (PMC8745465; doi:10.3390/ijms23010198)
Supplement: Supplementary file 1 [file ijms-23-00198-s001.zip › Table S5.pdf]

Table S5. The ratios of steroid hormones in the testes of control, diabetic and MF-treated diabetic rats, and the effect of a single dose of TP3 and hCG.

Group	Progesterone/ testosterone	17-0H-Progesterone/ testosterone	Androstenedione/ testosterone	Testosterone/ estradiol
C1	0.022 ± 0.002	4.06 ± 0.36	3.95 ± 0.20	0.306 ± 0.036
CT1	0.015 ± 0.001 [c]	2.21 ± 0.22 [c]	2.24 ± 0.25 [c]	0.760 ± 0.082 [c]
CG1	0.021 ± 0.001 [e]	1.92 ± 0.20 [d]	1.45 ± 0.08 [de]	1.749 ± 0.082 [de]
D1	0.042 ± 0.004 [a]	7.26 ± 0.51 [a]	8.75 ± 0.89 [a]	0.124 ± 0.016 [a]
DT1	0.019 ± 0.002 [c]	3.51 ± 0.56 [c]	2.67 ± 0.30 [c]	0.656 ± 0.104 [c]
DG1	0.022 ± 0.002 [d]	2.42 ± 0.08 [d]	1.91 ± 0.14 [d]	0.923 ± 0.075 [dg]
DM1	0.028 ± 0.002 [b]	5.10 ± 0.53 [b]	6.78 ± 0.51 [a]	0.281 ± 0.055 [b]
DMT1	0.011 ± 0.001 [ch]	2.17 ± 0.09 [ch]	2.78 ± 0.23 [c]	0.981 ± 0.102 [c]
DMG1	0.014 ± 0.001 [dg]	2.11 ± 0.13 [d]	1.95 ± 0.25 [d]	2.040 ± 0.162 [deh]

[a] – the difference between the C1 *vs*. D1 or DM1; [b] – the difference between the D1 *vs*. DM1; [c] – the difference between the C1 *vs*. CT1, D1 *vs*. DT1 and DM1 *vs*. DMT1; [d] – the difference between the C1 *vs*. CG1, D1 *vs*. DG1 and DM1 *vs*. DMG1; [e] – the difference between the CT1 *vs*. CG1 and DMT1 *vs*. DMG1; [g] – the difference between the CG1 *vs*. DG1 or DMG1; and [h] – the difference between the DT1 *vs*. DMT1 and DG1 *vs*. DMG1 are significant at $p<0.05$. The data are presented as the $M \pm SEM$, n=5.
